# Tumour‐macrophage crosstalk initiated by NFIC/METTL3 negative feedback loop via exosomal miR‐194‐5p promotes NSCLC progression

**DOI:** 10.1002/ctm2.70728

**Published:** 2026-06-30

**Authors:** Shu Fang, Mingyue Hao, Han Meng, Yuhang Jiang, Qiwen Li, Jiao Liang, Xiaolu He, Yi Hu, Linling Zhou, Qianrun Wang, Qiyuan Zhuo, Ji Wu, Kesong Shi

**Affiliations:** ^1^ Biomedical Research Institute Hubei Key Laboratory of Wudang Local Chinese Medicine Research Hubei University of Medicine Shiyan China; ^2^ Hubei Key Laboratory of Embryonic Stem Cell Research Hubei University of Medicine Shiyan China; ^3^ School of Basic Medical Sciences Hubei University of Medicine Shiyan China

**Keywords:** exosome, macrophage polarization, METTL3, miR‐194‐5p, NSCLC, ZNF106

## Abstract

**Background:**

The interplay between tumour cells and tumour‐associated macrophages (TAMs) within the tumour microenvironment is crucial for the progression of non‐small cell lung cancer (NSCLC). The underlying mechanisms involving RNA modification and exosomal communication remain incompletely understood.

**Methods:**

Multiplex immunofluorescence and flow cytometry were performed to evaluate M2 macrophage polarization. Exosomes were isolated by ultracentrifugation and validated by transmission electron microscopy, nanoparticle tracking analysis, and exosomal marker blots. To investigate the molecular mechanism, methylated RNA immunoprecipitation (MeRIP)‐qPCR and dual‐luciferase reporter assays were used to validate m^6^A modification sites on NFIC and miR‐194‐5p; RNA immunoprecipitation (RIP) confirmed the interaction between ZNF106 and interleukin‐6 (IL‐6) mRNA; chromatin immunoprecipitation (ChIP) was employed to detect STAT3 binding to the METTL3 promoter. The in vivo function of the identified feedback loop was assessed using an orthotopic xenograft mouse model of NSCLC.

**Results:**

A negative feedback loop between METTL3 and NFIC was demonstrated in NSCLC cells. METTL3 suppressed miR‐194‐5p expression and its loading into exosomes through m^6^A methylation. NSCLC‐derived exosomal miR‐194‐5p was internalized by macrophages and directly targeted ZNF106, thereby inhibiting M2 polarization. In macrophages, ZNF106 stabilized IL‐6 mRNA and promoted exosomal IL‐6 secretion, thereby activating the JAK2/STAT3 pathway and upregulating METTL3. This IL‐6‐driven METTL3 upregulation formed a positive feedback loop that sustains M2 polarization and tumour progression. In vivo disruption of this loop reduced tumour growth and metastasis.

**Conclusions:**

These findings establish a closed regulatory circuit initiated by an NFIC/METTL3 negative feedback loop. In this circuit, METTL3‐mediated m^6^A modification of exosomal miR‐194‐5p in NSCLC cells derepresses ZNF106 expression in macrophages, leading to IL‐6 production that activates the JAK2/STAT3 pathway and upregulates METTL3 in tumour cells, thereby perpetuating M2 polarization and malignant progression. This circuitry offers potential nodes for therapeutic intervention in NSCLC.

**Key points:**

1. NFIC/METTL3 negative feedback loop in NSCLC cells suppresses exosomal miR‐194‐5p via m6A methylation; reduced miR‐194‐5p deepresses ZNF106 in macrophages, promoting M2 polarization and IL‐6 secretion; Macrophage‐derived IL‐6 activates JAK2/STAT3 in NSCLC cells to upregulate METTL3, forming a positive feedback loop.

## Graphical abstract

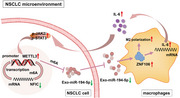

## 1 INTRODUCTION

Lung cancer ranks second in global cancer incidence. Non‐small cell lung cancer (NSCLC) represents approximately 84% of these cases.^1^ Although targeted and immunotherapies have advanced, the 5‐year survival rate for NSCLC remains around 25%,^2^ partly owing to poorly understood crosstalk between cancer cells and immune cells in the tumour microenvironment.^3^ Tumour‐associated macrophages (TAMs) are pivotal in this environment, influencing tumour dynamics, anti‐tumour immunity and immunotherapy responses, making them key therapeutic targets.^4^ Among them, M2‐polarized macrophages promote NSCLC progression by secreting immunosuppressive factors, facilitating angiogenesis, and remodelling the extracellular matrix.^5^ NSCLC cells, in turn, emit cytokines, exosomes and signalling molecules that drive macrophages toward the M2 phenotype, thereby establishing an autocrine–paracrine positive feedback loop between tumour cells and macrophages.^6^ Although the pro‐tumorigenic role of M2‐polarized TAMs in NSCLC is well‐documented, the precise mechanisms of microenvironmental remodelling through tumour and M2 macrophage interactions require further investigation.

METTL3, the primary m^6^A methyltransferase, exerts oncogenic functions in NSCLC primarily by mediating m^6^A modification of non‐coding RNAs^7^, ^8^, ^9^ or mRNAs.^10^ Furthermore, our previous study demonstrated that NFIC inhibits NSCLC progression by suppressing METTL3 transcription.^11^ However, the mechanism by which METTL3 regulates NFIC through m^6^A modification remains to be elucidated, particularly how this regulation participates in the crosstalk between lung cancer cells and TAMs and mediates microenvironmental remodelling.

Exosomes are pivotal in intercellular communication and cancer progression, notably in lung cancer.^12^ Exosome‐mediated information exchange establishes a crucial gene regulatory network governing bidirectional interactions between tumour cells and TAMs within the tumour microenvironment, profoundly affecting tumour growth, metastasis, immunosuppression and therapeutic resistance.^13^ Exosomes are enriched with diverse bioactive molecules, including proteins, miRNAs, lncRNAs and circRNAs, which exert important biological functions.^14^ For instance, exosomal miRNAs from various donor cells are linked to lung cancer pathogenesis and treatment.^15^ Recent studies have revealed that exosomes mediate communication between tumour cells and TAMs^13^; however, the detailed molecular mechanisms governing this process remain incompletely understood.

miRNA‐194, a crucial tumour regulator, has demonstrated tumour‐suppressive effects across various cancers by modulating key signalling pathways. Its expression levels are closely linked to patient prognosis and therapeutic outcomes. miRNA‐194 suppresses gastric cancer by targeting CCND1.^16^ In the context of NSCLC metastasis, exosomal lncRNA‐SOX2OT sponges miR‐194‐5p to promote bone metastasis via osteoclast differentiation.^17^ However, the mechanism by which m^6^A modification‐regulated exosomal miRNA‐194 mediates communication between tumour cells and macrophages remains largely unexplored.

This study explores the regulatory function of m^6^A modification via the NFIC/METTL3 feedback loop in exosomal miR‐194‐5p from NSCLC cells, aiming to clarify the molecular mechanisms that induce M2 macrophage polarization and facilitate tumour progression. We identify a negative feedback loop between NFIC and METTL3. METTL3 suppresses miR‐194‐5p expression and reduces the loading of miR‐194‐5p into exosomes through m^6^A methylation. NSCLC‐derived exosomal miR‐194‐5p is internalized by macrophages and directly targets ZNF106, thereby inhibiting M2 macrophage polarization. In macrophages, ZNF106 stabilizes IL‐6 mRNA and promotes exosomal IL‐6 secretion, which activates the JAK2/STAT3 pathway in NSCLC cells. Activation of the JAK2/STAT3 pathway upregulates METTL3 expression, forming a positive feedback loop. Thus, this study elucidates the tumour‐macrophage interaction mechanisms within the NSCLC microenvironment and suggests potential therapeutic strategies targeting this interaction for NSCLC treatment.

## 2 MATERIALS AND METHODS

### 2.1 Analysis of exosomal miRNA sequencing datasets from GEO

Exosomal miRNA sequencing datasets GSE114711 and GSE111803 (https://www.ncbi.nlm.nih.gov/geo) were downloaded from the Gene Expression Omnibus (GEO) database. GSE114711 comprises plasma‐derived exosomal small RNA sequencing data from 26 individuals, including 7 healthy smokers and 19 patients with NSCLC. GSE111803 comprises peripheral blood exosomal small RNA sequencing data from 5 patients with lung adenocarcinoma and 5 healthy controls. Raw sequencing data were analysed using the GEO2R pipeline, employing the limma package for differential expression analysis with criteria of *p* < .05 and |log2 fold change| > 1. Quality control metrics for each dataset were assessed as provided by the original submitters.

### 2.2 NSCLC tissue samples

Fifty‐five NSCLC tissues and forty‐three adjacent non‐cancerous tissues (HLugA098Bc01) were sourced from Outdo Biotech. This study involving human participants was approved by the Ethics Committee of Outdo Biotech (Approval No. YB M‐05‐02). Written informed consent was obtained from all participants, and all procedures performed during the study were in accordance with the principles of the Declaration of Helsinki. Adjacent non‐cancerous tissues were defined as lung parenchyma ≥ 3 cm from the tumour margin, with histologically confirmed absence of tumour cells. Detailed patient demographic and clinical information (including tissue type, sex, smoking history, pathological type and tumour location) is presented in Table S1.

### 2.3 Macrophage polarization and stable cell line construction

THP‐1 cells were exposed to phorbol 12‐myristate 13‐acetate (100 ng/mL, PMA, MCE) for 48 h to promote macrophage maturation. THP‐1‐derived macrophages were polarized into M1 or M2 phenotypes by culturing them for 96 h in medium supplemented with either lipopolysaccharide (LPS, 100 ng/mL; Solarbio) or interleukin‐4 (IL‐4, Proteintech), respectively. The procedures for METTL3 and NFIC knockdown or overexpression were as previously described in our published study.^11^ Stable cell lines with ZNF106 knockdown (sh‐ZNF106) or overexpression (oe‐ZNF106), along with their respective control groups, were constructed by GenePharma. Table S3 lists the shRNA sequences.

### 2.4 CRISPR/Cas9‐mediated gene knockout

Guide RNAs (gRNAs) targeting the METTL3 coding region were designed using Benchling and GenScript online tools to create METTL3 knockout cell lines. Refer to Table S2 for the oligonucleotide sequences used. The oligonucleotides were assembled into the lentiCRISPR v2 vector (Addgene, #52961) following standard protocols. The constructed plasmid was transfected into A549 and H460 cells at 60% confluence. Cells were treated with puromycin for 48 h after 36 h, followed by isolation of single‐cell clones through limiting dilution. Western blotting confirmed the knockout of METTL3.

### 2.5 Multiplex immunofluorescence and in situ hybridization

Primary antibodies against METTL3, ZNF106, CD68, CD206 and/or STAT3 were applied overnight at 4°C to tissue sections that had been deparaffinized, rehydrated, retrieved for antigen in citrate buffer (pH 6.0), and blocked with 5% normal goat serum for mIHC. Following incubation with horseradish peroxidase (HRP)‐conjugated secondary antibodies, tyramine signal amplification was performed using distinct fluorophores (Opal kits, Akoya Biosciences).

For in situ hybridization (ISH), the expression and subcellular localization of miR‐194‐5p were detected using a Cy3‐labelled locked nucleic acid (LNA) probe specific for miR‐194‐5p, which was generated by Gene‐Pharma Co., Ltd. with a Cy3 fluorophore at the 3′ end and LNA‐modified bases. After deparaffinization and protease digestion, sections were hybridized with the probe at 55°C overnight. Following stringent washes, the signal was visualized directly under a fluorescence microscope (Olympus FV3000RS).

For combined detection of miR‐194‐5p and METTL3 protein, sequential ISH and mIHC were performed, with ISH completed first, followed by the mIHC procedure as described above. Five random fields per section were quantified for positive cells using ImageJ.

### 2.6 Exosome isolation, tracking and co‐culture models

Exosomes were isolated from the indicated cells (1  ×  10^6^/well) after 48 h in vesicle‑depleted medium. The conditioned medium was cleared by low‑speed centrifugation (300× g, 2000× g, 10 000× g) and then ultracentrifuged at 100 000× g for 60 min to obtain exosome pellets. The pellet was resuspended in PBS and washed by another ultracentrifugation step at 100 000× g for 70 min at 4°C. The final exosome pellet was resuspended in 50 µL PBS and stored at ‐80°C for subsequent experiments. For TEM and nanoparticle tracking analysis (NTA), the exosome concentration was adjusted to 0.5 µg/µL. The morphology of isolated exosomes was examined using TEM with negative staining. Exosome size and concentration were assessed via NanoSight NS300 (Malvern) using NTA 3.0. For Western blot analysis of exosomal proteins, the final loading amount was 10 µg per lane.

PKH67/PKH26‑labeled exosomes (Sigma‐Aldrich) were prepared per the manufacturer's instructions and then added to unlabelled macrophages, A549, and H460 at 50 µg/mL for in vitro co‑culture. After co‐incubation at 37°C for 12 h, cells were visualized using a confocal laser scanning microscope (Olympus FV3000RS). For the in vivo mouse experiment, each mouse received 20 µg of exosomes (diluted in PBS, 100 µL per mouse) via tail vein injection.

### 2.7 Flow cytometry

Macrophage polarization was evaluated by blocking THP‐1‐derived macrophages with 5% normal goat serum for 15 min, then incubating them with PE‐conjugated anti‐human CD206 antibody (PE‐FcA98031, Proteintech) for 40 min at 4°C. Negative controls were implemented using isotype‐matched antibodies. After washing with PBS and resuspension in staining buffer, cells were analysed on a flow cytometer (BD Biosciences).

The following gating strategy was applied for all flow cytometry analyses of CD206^+^ macrophages. Cells were first gated on FSC‑A versus SSC‑A to remove debris and select the main population, then doublets were excluded via FSC‑H versus FSC‑A. Macrophages were then identified as CD68‐positive cells using PE‐conjugated anti‐human CD68 antibody. Finally, within the CD68^+^ gate, the percentage of CD206^+^ cells were quantified, with isotype controls used to set the threshold. This gating strategy was consistently applied across all flow cytometry experiments in this study.

### 2.8 qRT‐PCR and western blot analysis

Total RNA was isolated from cells using TRIzol (Invitrogen) and converted to cDNA using the PrimeScript RT Reagent Kit (Takara). The ABI 7500 Real‑Time PCR System (Applied Biosystems) was used for qRT‑PCR. Primer sequences are provided in Table S4. For Western blotting, cells were lysed in protease‑inhibitor‑supplemented RIPA buffer (Solarbio). Lysates containing equal protein amounts were resolved by 10% SDS‑PAGE and blotted onto 0.45 µm PVDF membranes (Servicebio). Membranes were blocked with 5% non‐fat milk for 1 h at room temperature, followed by incubation with primary antibodies overnight at 4°C and then with secondary antibodies for 2 h. Table S5 lists the primary and secondary antibodies used.

### 2.9 MeRIP‐qPCR and chromatin immunoprecipitation (ChIP)

MeRIP assays were performed with a commercial kit (P‐9018, EPIGENTEK) according to the manufacturer's protocol. In brief, total RNA was randomly fragmented into 100–200 nucleotide fragments, after which RNA was immunoprecipitated using an m^6^A‐specific antibody and subjected to qRT‐PCR.

For ChIP assays, the SimpleChIP Enzymatic Chromatin IP Kit (9003S, CST) was used. A549 and H460 cells were cross‐linked with 1% formaldehyde for 10 min to preserve DNA‐protein interactions. Following cell lysis and sonication, immunoprecipitation was performed using the indicated antibody. Precipitated chromatin DNA was then isolated and measured by qRT‑PCR.

### 2.10 Enzyme‐linked immunosorbent assay (ELISA)

Samples and serially diluted standards were loaded onto capture‑antibody‑coated 96‑well plates and held for 2 h at room temperature. The plates were then sequentially treated with biotinylated detection antibodies (1 h) and streptavidin–HRP conjugate (45 min). After adding tetramethylbenzidine substrate solution and a 15‑min incubation at room temperature, the reaction was stopped and absorbance read immediately at 450 nm (Thermo Fisher).

### 2.11 Orthotopic lung cancer model and immunohistochemistry (IHC)

The Animal Ethics Committee of Hubei University of Medicine approved all animal experiments (Approval No.: 03125110R). Four‐week‐old male BALB/c nude mice were anesthetized using 2% isoflurane. A549 cells (1 × 10^6^ cells) were mixed with indicated components (PBS, exosome‐pretreated macrophages, macrophages) and resuspended in 50 µL of a 1:1 mixture of RPMI medium and Matrigel. Each mouse received a direct injection of the cell suspension into the left lung. Tumour growth was monitored 4 weeks later using an IVIS Lumina II in vivo imaging system. After euthanasia, the left and right lungs were harvested, fixed and paraffin‑embedded for further analysis.

For IHC, tissue sections underwent deparaffinization, rehydration and antigen retrieval. They were then probed overnight at 4°C with primary antibodies against METTL3, p‑JAK2, p‑STAT3 and Ki67. After washing, the sections were exposed to biotinylated secondary antibodies for 1 h at room temperature. Signal was detected using a streptavidin–HRP conjugate and diaminobenzidine substrate. Images were captured using a microscope (Nikon).

### 2.12 Statistical analysis

Statistical analyses were conducted with GraphPad Prism 8.0.2 (GraphPad Software). All experiments included at least three biological replicates, with data shown as mean  ±  SD. Two‑group comparisons employed two‑tailed Student's *t*‑test, while multiple comparisons used one‑way ANOVA followed by Tukey's post‑hoc test.

Details on other materials and methods are provided in the Supporting Information.

## 3 RESULTS

### 3.1 METTL3/NFIC negative feedback loop regulates M2 macrophage polarization

**FIGURE 1:**
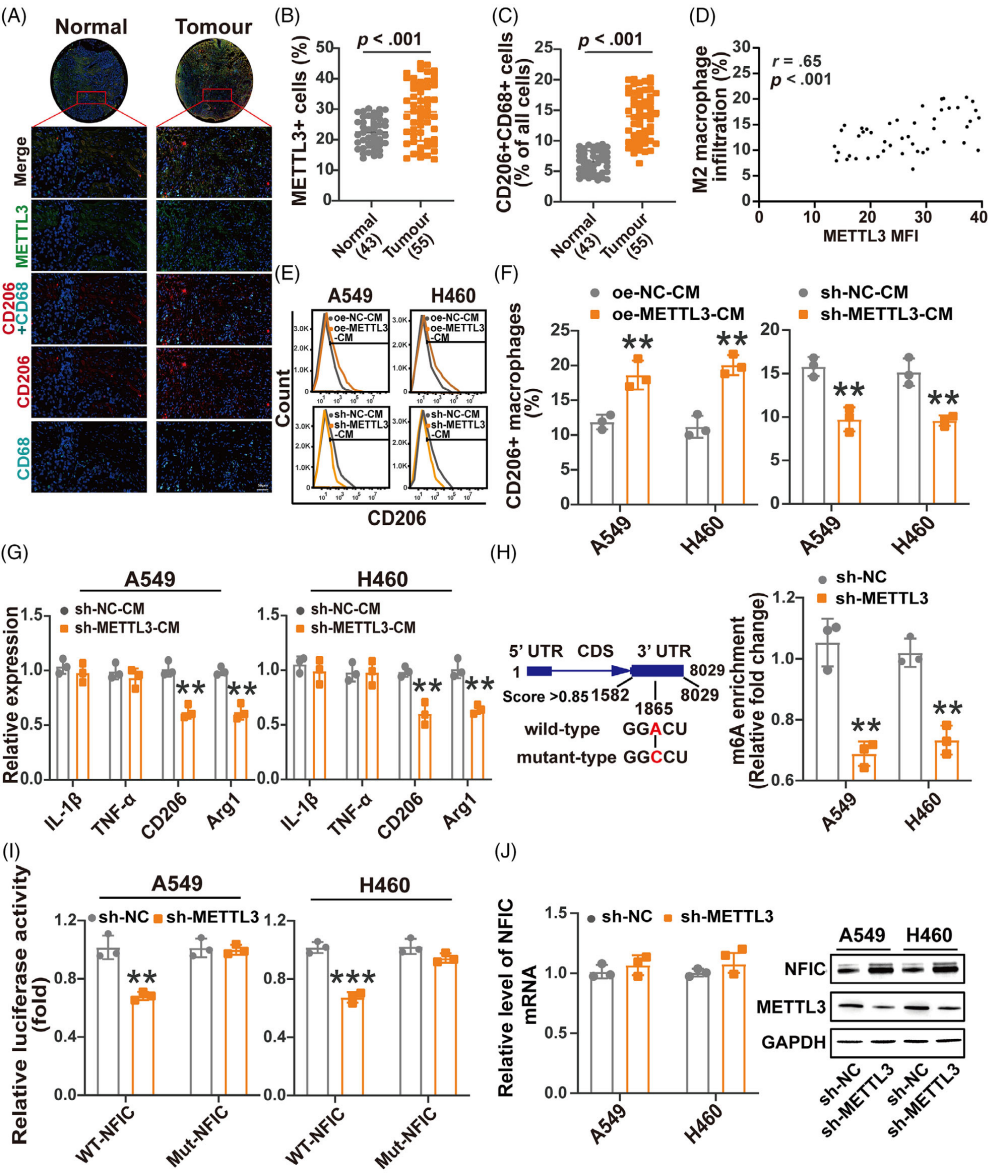
METTL3/NFIC negative feedback loop promotes M2 macrophage polarization in NSCLC. (A–C) Representative multiplex immunofluorescence staining images (A) and quantitative analysis of METTL3 expression (B) and CD206/CD68 positivity ratio (C) in NSCLC and adjacent normal tissues. Scale bar, 50 µm. (D) Correlation analysis between METTL3 expression level and CD206/CD68 positivity ratio in NSCLC tissues. (E and F) Flow cytometric analysis (E) and quantification (F) of CD206‐positive M0 macrophages stimulated with conditioned media from METTL3‐overexpressing or METTL3‐knockdown A549 and H460 cells. (G) Expression of M1 (IL‐1β, TNF‐α) and M2 (CD206, Arg1) macrophage markers was detected by qRT‐PCR. (H) Schematic diagram of the predicted m^6^A site within the NFIC 3′UTR and MeRIP‐qPCR analysis showing the effect of METTL3 knockdown on m^6^A enrichment at this site. (I) Dual‐luciferase reporter assay showing the effect of METTL3 knockdown on the luciferase activity of WT or Mut NFIC 3′UTR reporter. (J) Western blot and qRT‐PCR analysis of NFIC protein and mRNA expression following METTL3 knockdown. Data are presented as mean ± SD. **p < .01, ***p < .001.

To explore the role of METTL3 in macrophage polarization, we first used multiplex immunofluorescence to examine the correlation between METTL3 expression and M2 macrophage infiltration in NSCLC tissues. The results showed that both METTL3 expression levels and the CD206/CD68 positivity ratio were significantly elevated in NSCLC tissues compared with adjacent non‐cancerous tissues, indicating increased infiltration of M2 macrophages (Figure 1A–C). Correlation analysis further revealed a positive correlation between METTL3 expression and M2 macrophage infiltration (Figure 1D). In contrast, analysis of METTL3 expression versus M1 marker (CD86^+^) infiltration in the same NSCLC tissues showed no significant correlation (r = ‐.2309, *p* = .0759) (Figure S1A,B).

Conditioned media (CM) from A549 and H460 cells overexpressing METTL3 (oe‐METTL3‐CM) were used to stimulate M0‐type THP‐1 macrophages induced with phorbol 12‐myristate 13‐acetate (PMA) for 24 h. Compared with the control group (oe‐NC‐CM), stimulation with oe‐METTL3‐CM significantly increased the positivity rate of CD206 and the expression levels of M2 macrophage markers (Arg1 and CD206) in M0 macrophages (Figure 1E,F; Figure S1C). In contrast, CM from A549 and H460 cells with METTL3 knockdown (sh‐METTL3‐CM) produced opposite effects, while no significant changes were detected in the expression of M1 macrophage markers (IL‐1β and TNF‐α) (Figure 1E–G). Furthermore, treatment with CM from NFIC‐knockdown cells (sh‐NFIC‐CM) reversed the sh‐METTL3‐CM‐induced reduction in CD206 positivity and M2 marker expression (Figure S1D–F).

To uncover how METTL3 regulates NFIC, we first predicted potential m^6^A sites in NFIC using the SRAMP online tool. Analysis revealed a conserved m^6^A site within the 3′UTR (1865 bp) of NFIC mRNA (Figure 1H). This site was validated by MeRIP‑qPCR and dual‑luciferase reporter assays (Figure 1H,I). Meanwhile, METTL3 knockdown markedly increased NFIC protein expression without affecting its mRNA expression (Figure 1J).

To further confirm the specificity of METTL3 function, we generated METTL3 knockout (KO) A549 and H460 cells using CRISPR/Cas9 technology. Complete loss of METTL3 expression was confirmed by western blotting (Figure S2A). In METTL3 KO cells, NFIC protein expression was markedly increased (Figure S2A). Functional assays showed that METTL3 KO markedly inhibited A549 and H460 cell proliferation, migration, and invasion (Figure S2B–E). Additionally, CM from METTL3 KO A549 and H460 cells (KO‐METTL3‐CM) significantly inhibited the positivity rate of CD206 and the expression levels of M2 macrophage markers (Arg1 and CD206) in M0 macrophages (Figure S2F–H). These results are consistent with the shRNA‐mediated knockdown data and confirm that the observed phenotypes are specifically dependent on METTL3.

Based on our previous finding that NFIC transcriptionally represses METTL3 expression,^11^ these results indicate the existence of a METTL3/NFIC negative feedback loop, wherein NFIC inhibits METTL3 transcription, while METTL3 suppresses NFIC translation via m^6^A modification, thereby modulating M2 macrophage polarization through the secretory activity of NSCLC cells.

### 3.2 METTL3 promotes M2 macrophage polarization via NSCLC‐derived exosomal miR‐194‐5p

**FIGURE 2:**
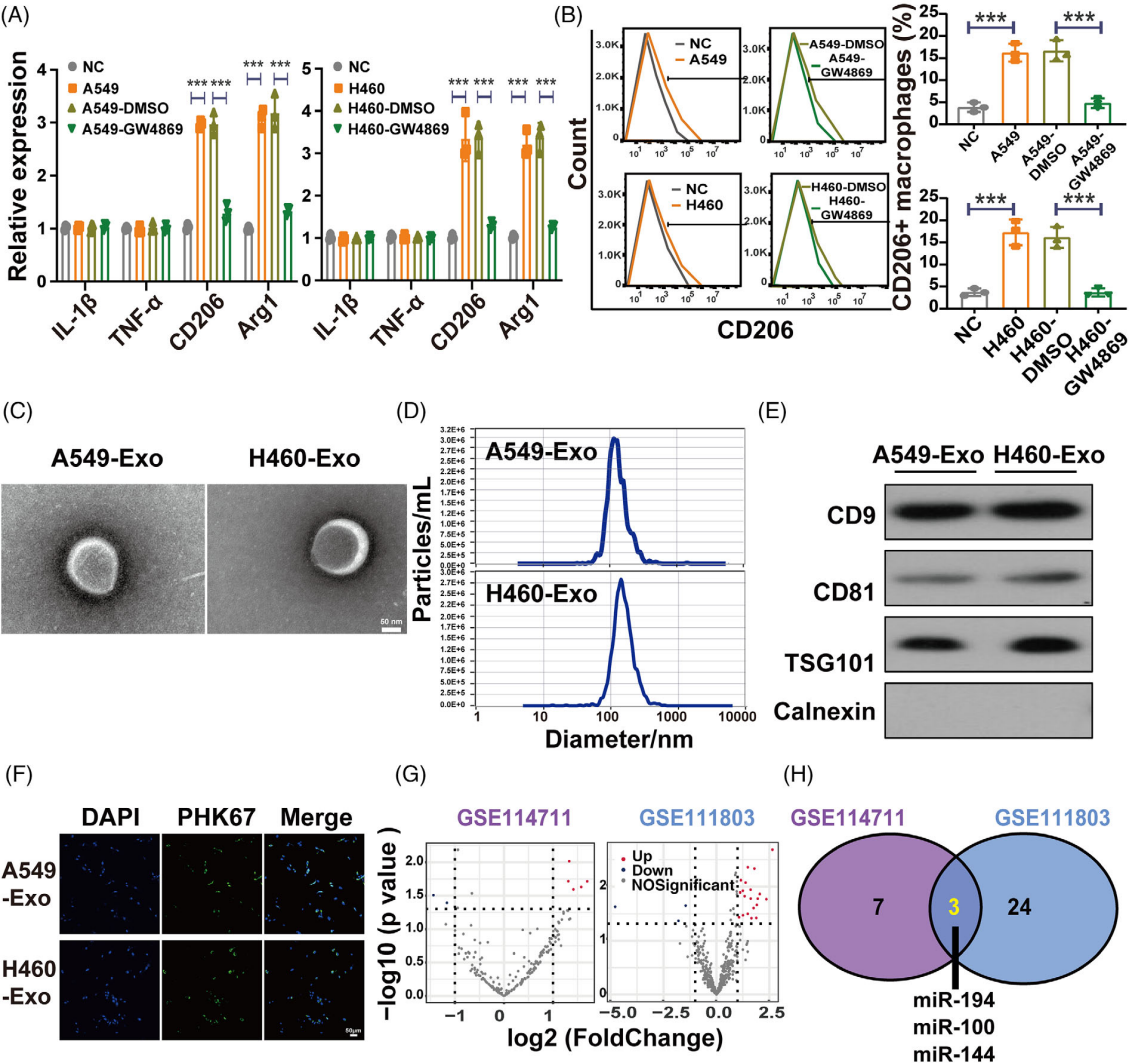
NSCLC cells induce M2 macrophage polarization in an exosome‐dependent manner. (A and B) qRT‐PCR (A) and Flow cytometric (B) analysis of CD206^+^ M0 macrophages stimulated with CM from A549 and H460 cells with or without GW4869 treatment. (C and D) Representative transmission electron microscopy images (C) and nanoparticle tracking analysis (D) of exosomes isolated from A549 and H460 cell culture supernatants. Scale bar, 50 nm. (E) Western blot analysis of exosomal marker proteins CD9, CD81 and TSG‐101 in exosomes. (F) Internalization of PKH67‐labelled exosomes derived from A549 and H460 cells by THP‐1 macrophages. Scale bar, 50 µm. (G) Volcano plots displaying differentially expressed exosomal miRNAs in the GSE114711 and GSE111803 datasets between NSCLC and normal lung tissues. Analysis of GEO‐derived exosomal miRNA‐seq datasets GSE114711 (*n* = 26: 7 healthy smokers, 19 NSCLC patients) and GSE111803 (*n* = 10: 5 lung adenocarcinoma patients, 5 healthy controls). (H) Venn diagrams showing the overlap between differentially expressed exosomal miRNAs in the two datasets. Data are presented as mean ± SD. ***p < .001.

To investigate whether METTL3 induces M2 macrophage polarization by regulating NSCLC‐derived exosomal miRNAs, CM from A549 and H460 cells were incubated with THP‐1 macrophages in the presence or absence of the exosome inhibitor GW4869. The CM from A549 and H460 cells increased the proportion of CD206^+^ macrophages (Figure 2A,B); however, treatment with GW4869 reversed the upregulation of M2 marker expression (CD206, Arg1), suggesting that NSCLC cells induce M2 macrophage polarization in an exosome‐dependent manner. Exosomes were subsequently isolated from the culture supernatant of NSCLC cells. Exosome morphology and size distribution were examined by transmission electron microscopy and nanoparticle tracking analysis, respectively (Figure 2C,D). Successful isolation was verified by Western blot detection of exosomal markers CD9, CD81 and TSG‐101 (Figure 2E). Furthermore, PKH67‐labelled exosomes derived from NSCLC (A549, H460) cells were taken up by macrophages after 24 h of co‑incubation (Figure 2F).

**FIGURE 3:**
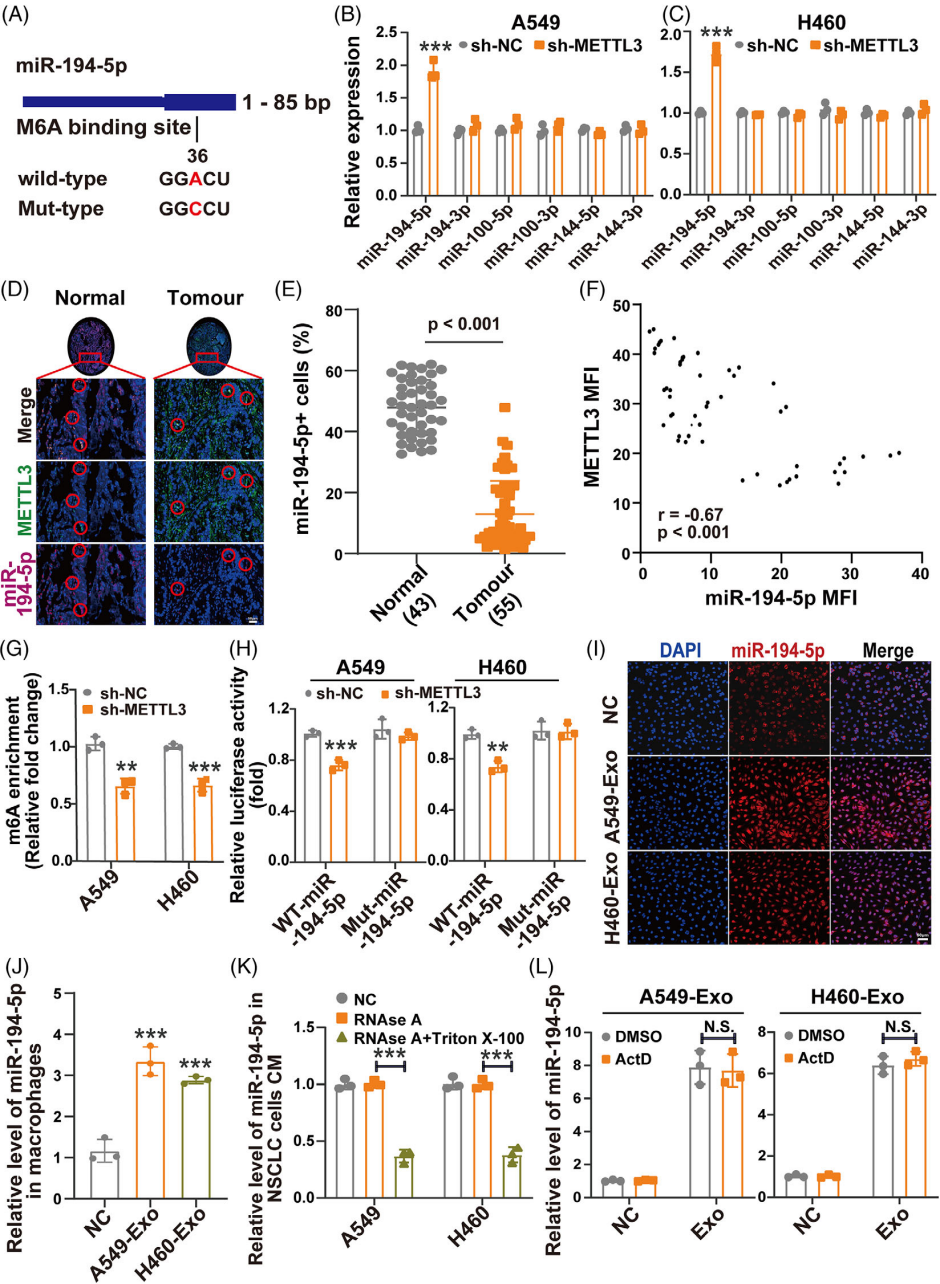
METTL3 regulates miR‐194‐5p expression and its exosomal transfer via m^6^A methylation in NSCLC cells. (A) Schematic diagram of the predicted m^6^A site within miR‐194‐5p. (B and C) qRT‐PCR analysis of miR‐194‐5p expression in A549 and H460 cells following METTL3 knockdown. (D) Representative multiplex immunofluorescence staining showing co‐localization of METTL3 and miR‐194‐5p in NSCLC tissues. Scale bar, 50 µm. (E) miR‐194‐5p expression in NSCLC and adjacent normal tissues. (F) Correlation analysis between METTL3 and miR‐194‐5p expression levels in NSCLC tissues. (G) MeRIP‐qPCR analysis showing the effect of METTL3 knockdown on m^6^A enrichment at the predicted site in miR‐194‐5p. (H) Dual‐luciferase reporter assay showing the effect of METTL3 knockdown on the luciferase activity of WT or Mut miR‐194‐5p. (I and J) In ISH (I) and qRT‐PCR (J) were used to detect the expression of miR‐194‐5p in THP‐1 macrophages after co‐incubation with exosomes derived from A549 and H460 cells. (K) qRT‐PCR analysis of miR‐194‐5p levels in the culture medium of A549 and H460 cells following treatment with RNase A and/or Triton X‐100. (L) qRT‐PCR analysis of miR‐194‐5p expression in macrophages co‐incubated with NSCLC‐derived exosomes in the presence or absence of ActD. Data are presented as mean ± SD. **p < .01, ***p < .001.

Analysis of exosomal miRNA‐seq datasets (GSE114711 and GSE111803) from NSCLC and normal lung tissues revealed 7 and 24 differentially expressed miRNAs in the GSE114711 and GSE111803 datasets, respectively, compared with normal lung tissue‐derived exosomal miRNAs (Figure 2G). Among these, miR‐194, miR‐100 and miR‐144 were commonly differentially expressed in both datasets (Figure 2H). The SRAMP online tool was used to predict potential m^6^A sites within these three shared miRNAs, revealing that only miR‐194‐5p (but not miR‐194‐3p) harbored an m^6^A site (Figure 3A). METTL3 knockdown significantly upregulated miR‐194‐5p expression in A549 and H460 cells (Figure 3B,C), whereas no significant changes were observed in the expression of miR‐100‐3p, miR‐100‐5p, miR‐144‐3p or miR‐144‐5p. Consistently, METTL3 knockout using CRISPR/Cas9 also led to a significant upregulation of miR‐194‐5p expression (Figure S3A). Additionally, miR‐194‐5p expression was downregulated in both NSCLC tissues and cells (Figure 3D,E; Figure S3B), and it co‐localized with METTL3 (Figure 3D), showing a significant negative correlation with METTL3 expression (Figure 3F).

Functional assays demonstrated that transfection with a miR‐194‐5p inhibitor significantly promoted the proliferation (Figure S3C,D), migration (Figure S3E,F) and invasion (Figure S3G,H) of A549 and H460 cells, whereas transfection with a miR‐194‐5p mimic produced opposite effects (Figure S3C–H). In addition, METTL3 overexpression significantly reversed the inhibitory effects mediated by the miR‐194‐5p mimic (Figure S4A–D). MeRIP‐qPCR revealed that METTL3 knockdown reduced m^6^A enrichment within the miR‑194‑5p fragment containing the predicted site (Figure 3G). Dual‑luciferase assays further demonstrated that METTL3 knockdown lowered wild‑type miR‑194‑5p reporter activity without affecting the mutant reporter (Figure 3H). Furthermore, when the m^6^A site mutant of miR‐194‐5p was used instead, the reversal effect of METTL3 on cell proliferation, migration, and invasion was completely abolished (Figure S4E,F). These findings indicate that METTL3 regulates miR‐194‐5p expression through m^6^A methylation in NSCLC cells, thereby modulating NSCLC progression.

To determine whether METTL3 regulates the loading of miR‐194‐5p into exosomes via m^6^A methylation to influence macrophage polarization, exosomes derived from A549 and H460 cells were incubated with macrophages. This resulted in a significant increase in miR‐194‐5p expression in macrophages (Figure 3I,J). In addition, treatment with RNase A combined with Triton X‐100 significantly reduced the levels of miR‐194‐5p in the culture medium of NSCLC cells (Figure 3K). Actinomycin D (ActD), an RNA transcription inhibitor, did not alter miR‐194‐5p levels in macrophages co‑incubated with NSCLC‑derived exosomes (Figure 3L). Moreover, exosomal miR‐194‐5p levels were reduced by METTL3 overexpression and elevated by METTL3 knockdown or knockout in NSCLC cells (Figure S4G). These results indicate that extracellular miR‐194‐5p is mainly packaged in membrane‐bound exosomes (not free) and is delivered from NSCLC cells to macrophages via these vesicles.

**FIGURE 4:**
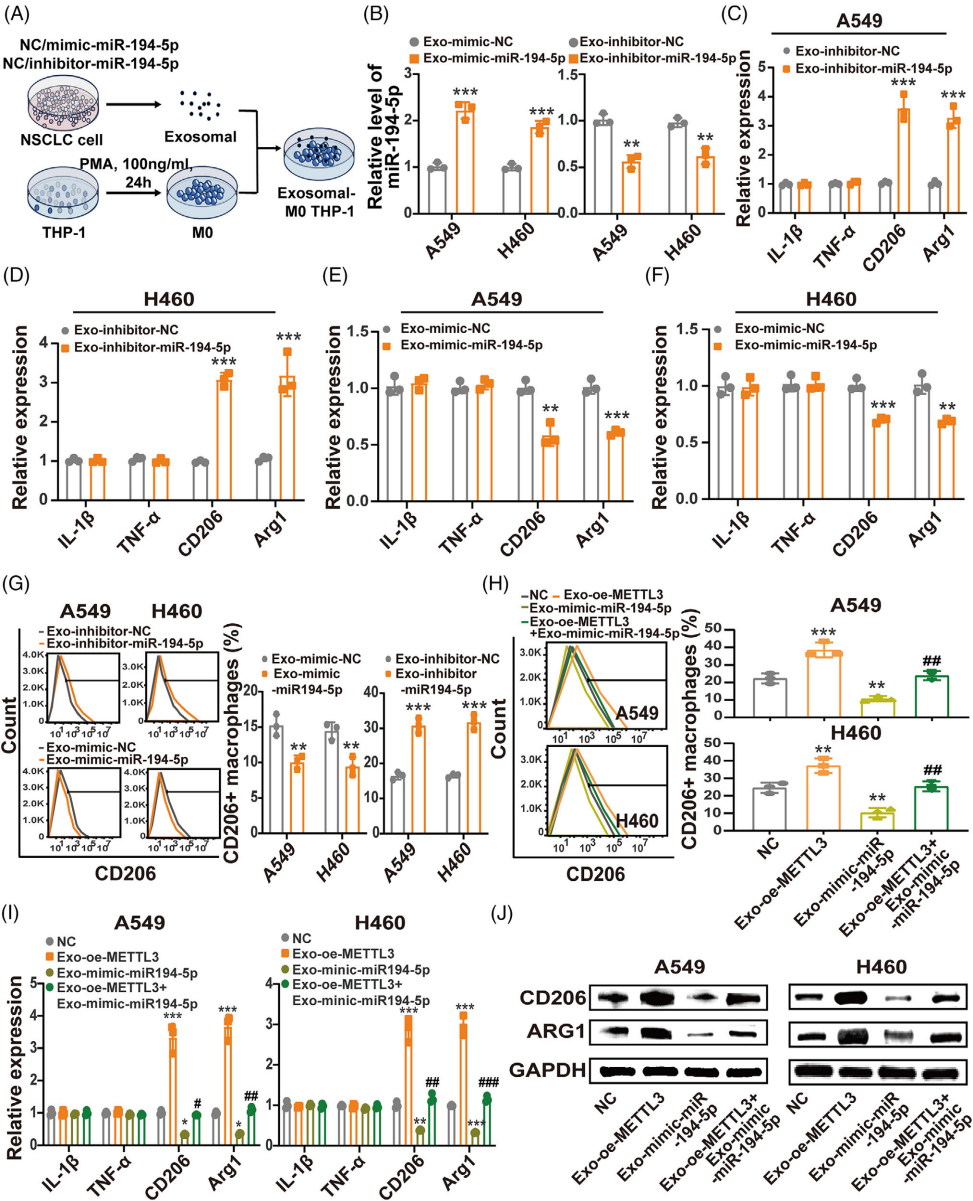
METTL3‐mediated m^6^A methylation of miR‐194‐5p inhibits M2 macrophage polarization by reducing its exosomal loading. (A and B) Schematic illustration of the experimental design and miR‐194‐5p expression in THP‐1 macrophages. (C–F) qRT‐PCR analysis of M2 (CD206, Arg1) and M1 (IL‐1β, TNF‐α) marker expression in macrophages treated with Exo‐inhibitor‐miR‐194‐5p (C‐D) or Exo‐mimic‐miR‐194‐5p (E‐F). (G and H) Flow cytometric analysis of CD206^+^ M0 macrophages. (I) qRT‐PCR analysis of M1 (IL‐1β, TNF‐α) and M2 (CD206, Arg1) marker expression. (J) Western blot analysis of M2 (CD206, Arg1) marker expression. Data are presented as mean ± SD. **p < .01, ***p < .001, compared with the NC group; ^#^ *p* < .05, ^##^ *p* < .01, ^###^ *p* < .001, compared with the Exo‐minic‐miR194‐5p group.

NSCLC cells were transfected with a miR‐194‐5p inhibitor or mimic, and the corresponding exosomes (Exo‐inhibitor‐miR‐194‐5p or Exo‐mimic‐miR‐194‐5p) were collected and co‐incubated with macrophages. Exo‐inhibitor‐miR‐194‐5p treatment led to decreased miR‐194‐5p expression in macrophages, whereas Exo‐mimic‐miR‐194‐5p treatment resulted in a significant increase (Figure 4A,B). Moreover, Exo‐inhibitor‐miR‐194‐5p treatment significantly upregulated the expression of M2 marker genes (CD206, Arg1) and the proportion of CD206^+^ macrophages, whereas Exo‐mimic‐miR‐194‐5p treatment exerted opposite effects, with no significant changes in M1 marker expression observed in either group (Figure 4C–G). Furthermore, exosomes derived from NSCLC cells overexpressing METTL3 reversed the upregulation of M2 marker gene expression and the increase in CD206^+^ macrophage proportion (Figure 4H–J). As additional controls, exosomes collected from normal human lung epithelial HBE cells were co‐incubated with macrophages. As shown in Figures S4H and S5A, HBE‑derived exosomes did not significantly alter the percentage of CD206^+^ macrophages or the expression of M2 marker genes, confirming the specificity of NSCLC‑derived exosomes. Moreover, we verified that transfection with the miR‐194‐5p inhibitor or mimic did not affect exosome yield (Figure S5B). Furthermore, a non‑exosome control group (the final supernatant after ultracentrifugation, exosome‑depleted medium) was included; as shown in Figure S5C, the exosome‑depleted medium did not elevate miR‐194‐5p levels in macrophages, confirming that the observed miR‐194‐5p transfer was indeed exosome‑dependent. These results indicate that METTL3 suppresses miR‐194‐5p expression via m^6^A methylation in NSCLC cells, thereby reducing its loading into exosomes and promoting M2 macrophage polarization. The molecular mechanism by which NSCLC‐derived exosomal miR‐194‐5p enters macrophages and regulates M2 polarization warrants further investigation.

### 3.3 NSCLC‐derived exosomal miR‐194‐5p inhibits M2 macrophage polarization by targeting ZNF106

**FIGURE 5:**
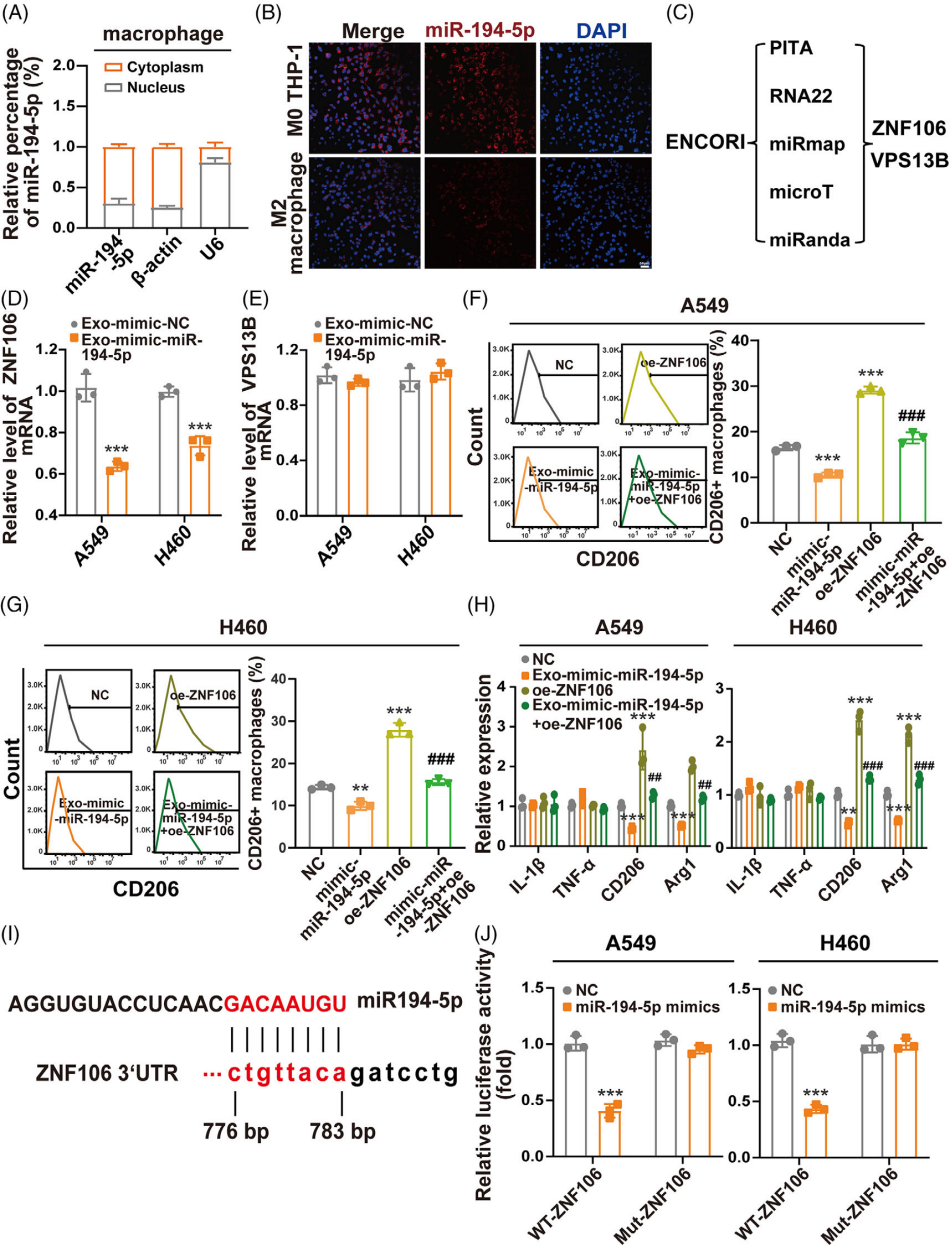
NSCLC‐derived exosomal miR‐194‐5p inhibits M2 macrophage polarization by targeting ZNF106. (A) The subcellular localization analysis of miR‐194‐5p in macrophages by nuclear‐cytoplasmic fractionation. (B) ISH showing the subcellular localization and expression of miR‐194‐5p in M0 and M2 macrophages. Scale bar, 50 µm. (C) The overlap of predicted miR‐194‐5p target genes from five algorithms (PITA, RNA22, miRmap, microT and miRanda) in the ENCORI database. (D and E) qRT‐PCR analysis of *ZNF106* and *VPS13B* mRNA expression in macrophages. (F and G) Flow cytometric analysis of CD206^+^ macrophages. (H) qRT‐PCR analysis of M1 (IL‐1β, TNF‐a) and M2 marker (CD206, Arg1) expression in macrophages. (I) Schematic diagram showing the predicted miR‐194‐5p binding site within the *ZNF106* 3′UTR. (J) Dual‐luciferase reporter assay showing the effect of miR‐194‐5p mimic on the luciferase activity of WT or Mut *ZNF106* 3′UTR reporter. Data are presented as mean ± SD. **p < .01, ***p < .001, compared with the NC group; ^##^ *p* < .01, ^###^ *p* < .001, compared with the oe‐ZNF106 group.

To explore how NSCLC‑derived exosomal miR‑194‑5p controls M2 macrophage polarization, we first examined its subcellular localization in macrophages. The results showed that miR‐194‐5p was predominantly localized in the cytoplasm (Figure 5A,B), suggesting that it may function by targeting mRNAs. In addition, ISH revealed that miR‐194‐5p expression was lower in M2 macrophages compared with M0 macrophages (Figure 5B). Potential target genes of miR‐194‐5p were predicted using the ENCORI database (integrating PITA, RNA22, miRmap, microT, and miRanda algorithms). *ZNF106* and *VPS13B* were identified as candidate target genes (Figure 5C). Macrophages treated with exosomes from miR‑194‑5p mimic‑transfected NSCLC cells (Exo‑mimic‑miR‑194‑5p) showed reduced ZNF106 mRNA and protein levels, while VPS13B mRNA remained unchanged (Figure 5D,E; Figure S5D). Furthermore, ZNF106 was upregulated in NSCLC tissues versus adjacent normal tissues and positively correlated with M2 macrophage infiltration (Figure S5E–G). Knockdown of ZNF106 in macrophages significantly decreased the proportion of CD206^+^ cells and the expression of M2 macrophage markers, whereas ZNF106 overexpression produced opposite effects (Figure S5H; Figure S6A–C). Notably, Exo‐mimic‐miR‐194‐5p reversed the enhanced M2 macrophage polarization induced by ZNF106 overexpression (Figure 5F–H). Bioinformatics analysis revealed a putative binding site for miR‐194‐5p within the 3′UTR of ZNF106 (Figure 5I). Dual‐luciferase reporter assays further confirmed that miR‐194‐5p directly bound to the 3′UTR of ZNF106 and suppressed its luciferase activity, whereas no significant effect was observed on the mutant construct (Figure 5J). Collectively, these results demonstrate that NSCLC‐derived exosomal miR‐194‐5p inhibits M2 macrophage polarization by targeting ZNF106.

### 3.4 Macrophage ZNF106 promotes NSCLC progression via exosomal IL‐6

**FIGURE 6:**
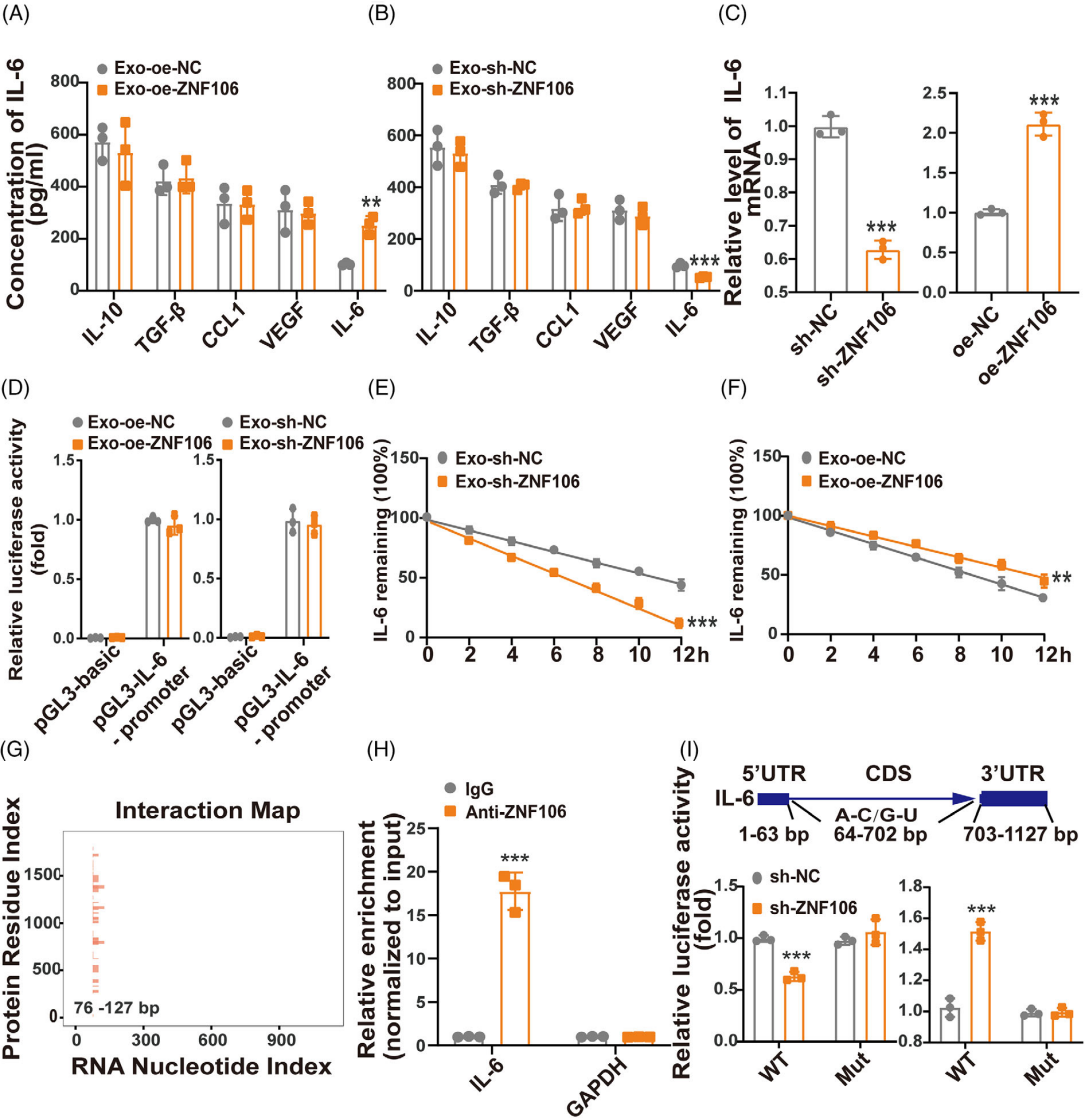
ZNF106 enhances *IL‐6* mRNA stability and promotes exosomal IL‐6 secretion in macrophages. (A–C) ELISA (A and B) and qRT‑PCR (C) analysis of indicated cytokine levels in macrophage‑derived exosomes following ZNF106 overexpression or knockdown. (D) ChIP‑qPCR analysis showing the binding of ZNF106 to the IL‑6 promoter region. (E and F) IL‑6 mRNA half‑life after ZNF106 knockdown (E) or overexpression (F) in macrophages treated with ActD. (G) Schematic diagram of the predicted ZNF106 binding site within the *IL‑6* mRNA (76–127 bp) based on the catRAPID database. (H) RIP assay confirming the direct interaction between ZNF106 and *IL‑6* mRNA. (I) Dual‑luciferase reporter assay showing the effect of ZNF106 on the luciferase activity of WT or Mut *IL‑6* mRNA reporter constructs. Data are presented as mean ± SD. **p < .01, ***p < .001.

Having established that NSCLC‐derived exosomal miR‐194‐5p suppresses M2 polarization by targeting ZNF106 in macrophages, we next investigated whether ZNF106‐induced M2 macrophages could, in turn, reciprocally regulate the malignant progression of NSCLC cells. Given the well‐established crosstalk between tumour cells and macrophages,^18^ exosomes were isolated from the CM of macrophages overexpressing ZNF106 (Exo‑oe‑ZNF106) and then co‑incubated with NSCLC cells. Exo‑oe‑ZNF106 enhanced the proliferation, migration, and invasion of A549 and H460 cells (Figure S6D–F). To clarify how macrophage ZNF106 drives NSCLC cell progression, we screened for potential cytokine mediators. Previous studies have shown that M2‑polarized macrophages secrete various cytokines, including IL‑10, TGF‑β, CCL1, VEGF and IL‑6, to promote NSCLC progression.^19^, ^20^ Given the RNA‐binding property of ZNF106, we hypothesized that it might regulate the expression of specific cytokines within exosomes. ELISA measured cytokine levels in macrophage‑derived exosomes after ZNF106 overexpression or knockdown. The results showed that ZNF106 overexpression specifically increased IL‐6 secretion and mRNA expression in exosomes, whereas ZNF106 knockdown exerted the opposite effect (Figure 6A–C). Notably, ZNF106 did not bind to the IL‐6 promoter region (Figure 6D), suggesting a post‐transcriptional regulatory mechanism.

To elucidate the mechanism by which ZNF106 regulates IL‐6 expression, the RNA polymerase inhibitor ActD was used to block mRNA synthesis. ZNF106 knockdown significantly shortened the half‐life of IL‐6 mRNA, whereas ZNF106 overexpression markedly prolonged it (Figure 6E,F). The catRAPID database predicted a potential ZNF106 binding site within the 76–127 bp region of IL‐6 mRNA (Figure 6G). RIP assay confirmed a direct interaction between ZNF106 and IL‐6 mRNA (Figure 6H). Dual‐luciferase reporter assays further demonstrated that ZNF106 directly bound to IL‐6 mRNA and enhanced its stability, whereas no significant effect was observed on the mutant construct (Figure 6I). Collectively, these findings suggest that ZNF106 regulates *IL‐6* mRNA stability by binding to its transcript.

**FIGURE 7:**
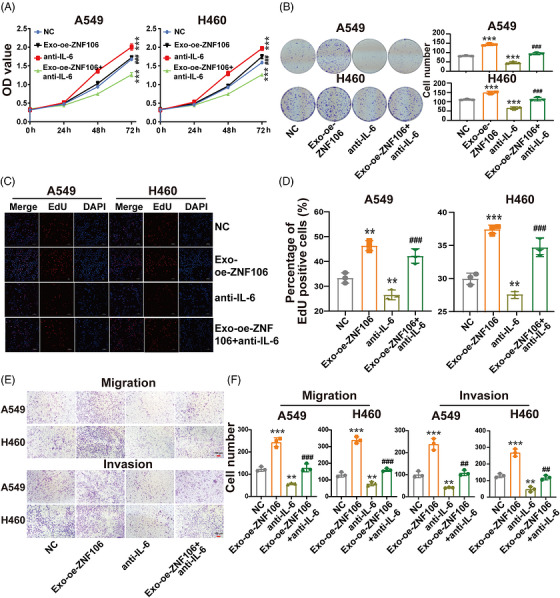
Macrophage‐derived exosomal IL‐6 mediates ZNF106‐induced promotion of NSCLC cell progression. (A–D) CCK‐8 (A), colony formation (B), and EdU assay (C and D) showing the proliferation of A549 and H460 cells after co‐incubation with macrophage‐derived Exo‐oe‐ZNF106 in the presence or absence of IL‐6 neutralizing antibody (anti‐IL‐6). (E and F) Migration and invasion of A549 and H460 cells assessed by transwell assay. Scale bar, 100 µm. Data are presented as mean ± SD. **p < .01, ***p < .001, compared with the NC group; ^##^ *p* < .01, ^###^ *p *< .001, compared with the anti‐IL‐6 group.

Furthermore, co‐incubation of NSCLC cells with Exo‐oe‐ZNF106 derived from macrophages significantly promoted the proliferation (Figure 7A–D), migration (Figure 7E,F), and invasion (Figure 7E,F) of A549 and H460 cells, and these effects were strongly attenuated by the addition of an IL‐6 neutralizing antibody (Figure 7A–F). In conclusion, macrophage ZNF106 enhances IL‐6 mRNA stability, promotes exosomal IL‐6 secretion, and thereby drives the malignant progression of NSCLC cells.

### 3.5 M2 macrophage‐derived il‐6 upregulates METTL3 expression in NSCLC cells via activation of the JAK2/STAT3 signalling pathway

**FIGURE 8:**
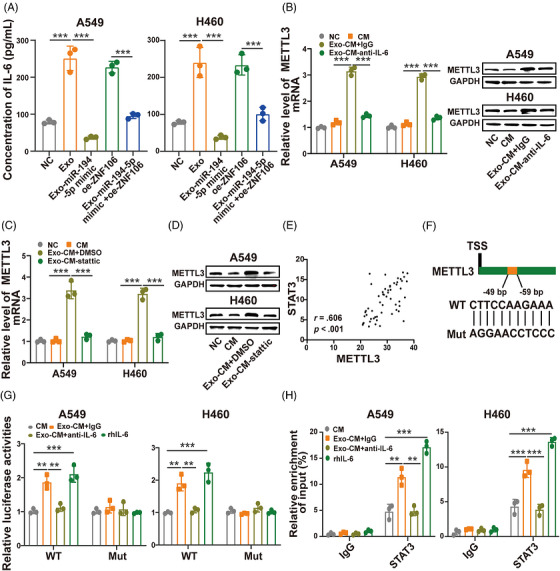
M2 macrophage‐derived IL‐6 upregulates METTL3 expression in NSCLC cells via activation of the JAK2/STAT3 signaling pathway. (A) ELISA analysis of IL‐6 expression. (B) qRT‐PCR analysis of METTL3 expression in NSCLC cells co‐cultured with Exo‐CM. (C‐D) qRT‐PCR (C) and western blot (D) analysis of METTL3 expression in A549 and H460 cells treated with Exo‐CM with or without the STAT3 inhibitor Stattic. (E) Correlation analysis between METTL3 and STAT3 expression in NSCLC tissues. (F) Schematic diagram showing the predicted STAT3 binding sites within the METTL3 promoter region based on the JASPAR database. (G) Dual‐luciferase reporter assay showing the effect of Exo‐CM on the luciferase activity of WT or Mut METTL3 promoter constructs. (H) ChIP‐qPCR analysis of STAT3 enrichment at the METTL3 promoter region in A549 and H460 cells treated with Exo‐CM. Data are presented as mean ± SD. **p < .01, ***p < .001.

Previous studies have shown that M2 macrophages can upregulate METTL3 expression in hepatocellular carcinoma cells via the exosomal IL‐6/STAT3 signalling pathway.^21^ Therefore, we further investigated whether a similar regulatory mechanism exists in NSCLC. Notably, co‐culturing A549 and H460 cells with exosomes from macrophages exposed to NSCLC cell‐derived exosomes (Exo‐CM) resulted in elevated IL‐6 and METTL3 expression. Exosomes from macrophages exposed to NSCLC cell‐derived exosomes with miR‐194‐5p mimics (Exo‐mimic‐CM) significantly decreased IL‐6 expression in co‐cultured NSCLC cells, an effect reversed by ZNF106 overexpression (Figure 8A,B). The introduction of an IL‐6 neutralizing antibody to the co‐culture system significantly inhibited the Exo‐CM‐induced increase of METTL3 in A549 and H460 cells (Figure 8B).

Given that JAK2/STAT3 is a key downstream effector pathway of IL‐6 signaling,^22^, ^23^ we next explored whether IL‐6 promotes METTL3 expression through activation of the JAK2/STAT3 axis. The results showed that co‐culture with Exo‐CM significantly increased the phosphorylation levels of JAK2 and STAT3 in NSCLC cells, whereas the addition of an IL‐6 antibody substantially inhibited this effect (Figure S6G). Moreover, the STAT3 inhibitor Stattic reversed the Exo‑CM‑induced increase in METTL3 expression in A549 and H460 cells (Figure 8C,D). In NSCLC tissues, METTL3 and STAT3 expression levels were positively associated (Figures 8E and S7H). JASPAR database analysis predicted potential STAT3 binding sites in the METTL3 promoter region (Figure 8F). ChIP assays confirmed that the STAT3 antibody significantly enriched the binding site (located at ‐49 to ‐59 bp upstream of the promoter) (Figure S6I). The dual‐luciferase reporter assay results showed that Exo‐CM significantly enhanced the luciferase activity of the WT promoter but had no significant effect on the Mut promoter. Moreover, the addition of exogenous IL‐6 antibody attenuated the Exo‐CM‐induced increase in luciferase activity (Figure 8G). ChIP assays further verified that Exo‐CM treatment enhanced STAT3 enrichment at the METTL3 promoter region, and this enrichment was suppressed by IL‐6 antibody treatment (Figure 8H). Collectively, these results suggest that M2 macrophage‑derived IL‑6 elevates METTL3 expression in NSCLC cells via JAK2/STAT3 activation, creating a positive feedback loop.

### 3.6 In vivo validation and schematic model of the mechanism

**FIGURE 9:**
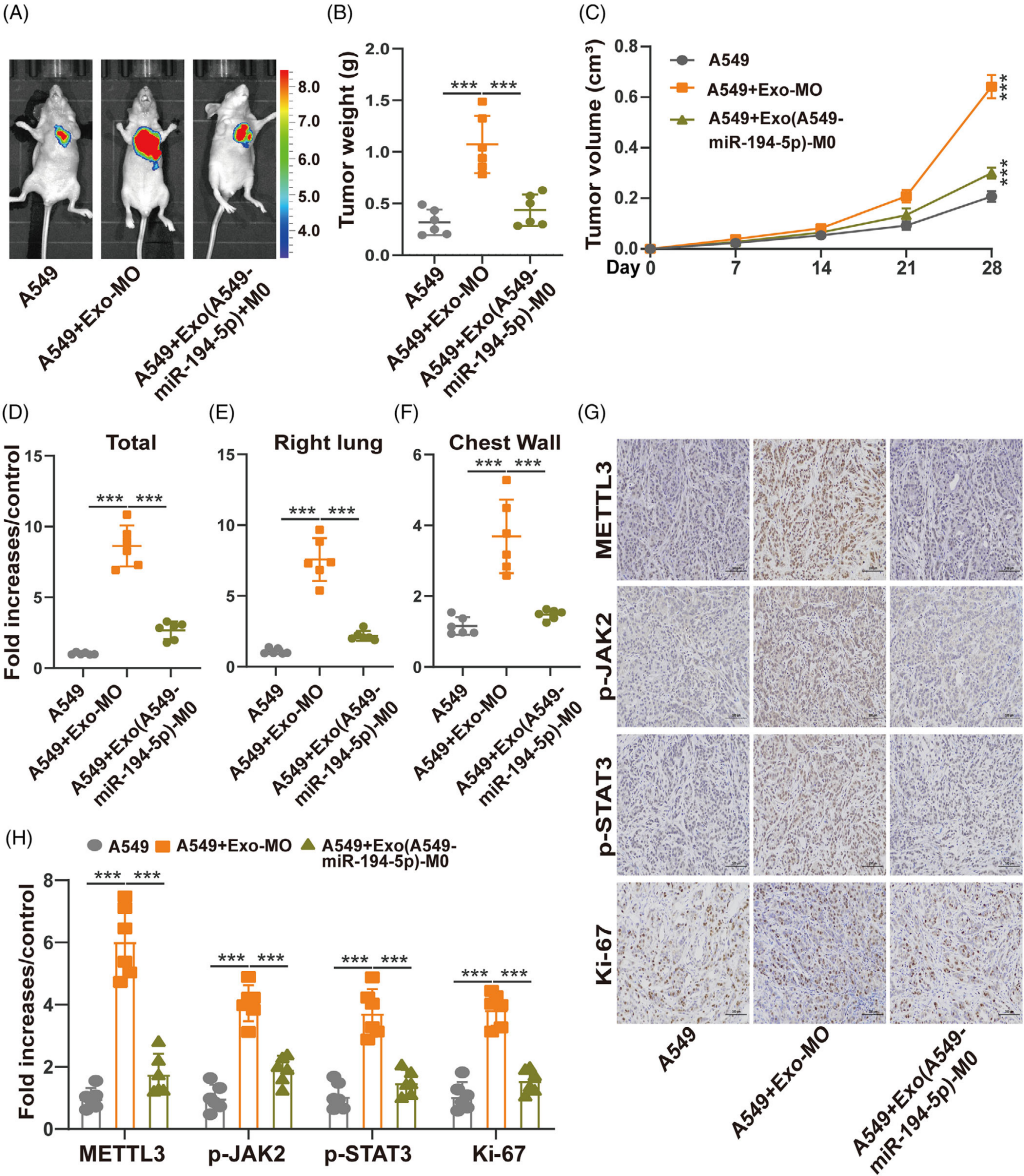
NSCLC exosome‐induced M2 macrophages promote tumour growth and metastasis in vivo. (A–D) Representative bioluminescence images (A), and tumour weight (B), tumour volume (C) and quantification of photon flux (D) of mice in each group. (E and F) Metastatic signals in the contralateral lung (right lung, E) and chest wall (F). (G and H) Representative immunohistochemistry staining (G) and quantification (H) of METTL3, p‐JAK2, p‐STAT3, and Ki67 expression in tumour tissues. Scale bar, 50 µm. Data are presented as mean ± SD. **p < .01, ***p < .001.

We established an orthotopic xenograft mouse model to further assess the impact of NSCLC exosome‑induced M2 macrophages on NSCLC cell proliferation and metastasis. A549 cells were mixed with the following components and orthotopically implanted into the left lung: PBS (A549 alone group); M0 macrophages pre‐treated with NSCLC cell‐derived exosomes (Exo‐M0); M0 macrophages pre‐treated with NSCLC cell‐derived exosomes transfected with miR‐194‐5p mimics (Exo‐mimic‐M0); M2 macrophages with negative control siRNA (M2/sh‐NC); and M2 macrophages with IL‐6 siRNA (M2/sh‐IL‐6) (Figure 9A; Figure S7A). The results revealed that, relative to the A549 alone group, the groups co‐implanted with Exo‐M0 or M2/sh‐NC exhibited larger tumour volumes, greater tumour weights, and more pronounced metastatic signals in the contralateral lung (right lung) and chest wall (Figure 9B–F; Figure S7B–F). In contrast, the Exo‐mimic‐M0 and M2/sh‐IL‐6 groups showed significantly reduced tumour volumes and weights (Figure 9B–F; Figure S7B–F). Immunohistochemical (IHC) staining further confirmed that tumour tissues from mice injected with A549 cells mixed with Exo‐M0 or M2/sh‐NC had significantly elevated expression levels of METTL3, p‐JAK2, p‐STAT3 and Ki67, whereas the Exo‐mimic‐M0 and M2/sh‐IL‐6 groups showed significantly restored levels of these proteins to values comparable to those of the A549 alone group (Figure 9G,H; Figure S7G‐H). Furthermore, multiplex immunofluorescence staining for p‐STAT3 and the tumour cell marker Pan‐CK revealed that, relative to the A549 alone group, the A549 + M2/sh‐NC group had a significantly higher percentage of p‐STAT3^+^/Pan‐CK^+^ double‐positive cells among total Pan‐CK^+^ tumour cells. This increase was significantly reversed in the A549 + M2/sh‐IL‐6 group (Figure S7I,J), indicating that the enhanced p‐STAT3 signal originates primarily from tumour cell.

**FIGURE 10:**
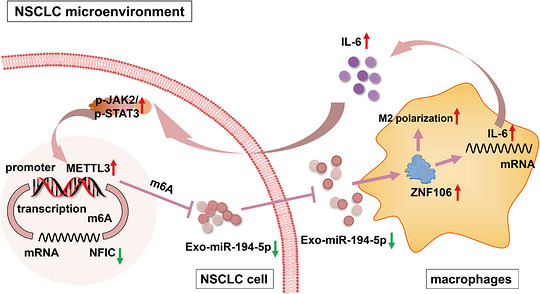
Schematic model of the proposed tumour‐macrophage crosstalk mechanism in NSCLC. NFIC/METTL3 negative feedback loop in NSCLC cells suppresses exosomal miR‐194‐5p via m^6^A methylation; Reduced miR‐194‐5p deepresses ZNF106 in macrophages, promoting M2 polarization and IL‐6 secretion; Macrophage‐derived IL‐6 activates JAK2/STAT3 in NSCLC cells to upregulate METTL3, forming a positive feedback loop.

This study elucidates the crosstalk mechanism between M2 macrophages and tumour cells in the NSCLC microenvironment. In NSCLC cells, NFIC transcriptionally represses METTL3 expression, while METTL3 suppresses NFIC translation via m^6^A methylation, forming an NFIC/METTL3 negative feedback loop. Subsequently, METTL3 in NSCLC cells suppresses exosomal miR‐194‐5p expression via m^6^A modification, leading to reduced miR‐194‐5p levels in secreted exosomes. The downregulation of exosomal miR‐194‐5p relieves its inhibition on the RNA‐binding protein ZNF106 in macrophages, thereby upregulating ZNF106 expression and promoting M2 macrophage activation. These activated M2 macrophages secrete IL‐6, which acts on adjacent NSCLC cells to facilitate their malignant progression. Moreover, IL‐6 triggers JAK2/STAT3 signalling, leading to METTL3 upregulation in NSCLC cells and thereby creating a positive feedback loop (Figure 10).

## 4 DISCUSSION

In this study, we identified a tumour–macrophage crosstalk network mediated by the METTL3/miR‐194‐5p/ZNF106/IL‐6 axis in NSCLC. In this mechanism, m^6^A modification reprogrammes tumour‐derived exosomal miRNAs, leading to M2 macrophage polarization and creating a positive feedback loop that drives malignant progression. Our findings provide new molecular insights into tumour‐immune cell interactions within the NSCLC microenvironment.

Initially, METTL3 expression was found to positively correlate with M2 macrophage infiltration in NSCLC tissues. Our previous work demonstrated that NFIC suppresses NSCLC progression by inhibiting METTL3 transcription.^11^ Additionally, a study reported that METTL3 regulates NFIC expression via m^6^A modification in apical periodontitis.^24^ Based on these findings, we hypothesized that METTL3 may similarly regulate NFIC expression through m^6^A modification in NSCLC. Experimental results confirmed that METTL3 suppresses NFIC translation via m^6^A methylation, while NFIC transcriptionally inhibits METTL3 expression, forming a negative feedback loop that regulates NSCLC progression and promotes M2 macrophage polarization. This study further uncovers a novel function of this negative feedback loop in tumour immune microenvironment remodelling.

Exosomes critically mediate intercellular communication and remodel the tumour microenvironment by selectively packaging miRNAs that act as pro‑tumour messengers.^25^ In this study, through analysis of exosomal miRNA‐seq data from NSCLC and normal lung tissues, we identified miR‐194‐5p as a differentially expressed miRNA. Further experiments showed that NSCLC cells deliver miR‐194‐5p to macrophages via exosomes, suppressing M2 polarization. As a tumour suppressor, miR‐194‐5p regulates key signalling pathways in multiple cancers, and its expression correlates with gastric cancer patient prognosis.^16^ Current evidence emphasizes lncRNA‐mediated regulation, with tumour‑derived exosomal lncRNA‐SOX2OT acting as a miR‐194‐5p sponge to drive bone metastasis.^17^ However, the regulatory mechanism of miR‐194‐5p in TAMs and its m^6^A modification have not been reported. In this study, we identified an m^6^A modification site within miR‐194‐5p and demonstrated that METTL3 suppresses its expression and exosomal loading in NSCLC cells via m^6^A methylation, thereby promoting M2 macrophage polarization.

Further mechanistic investigation revealed that NSCLC‐derived exosomal miR‐194‐5p, upon entering macrophages, directly targets the 3′UTR of ZNF106 and inhibits its expression. ZNF106 is an RNA‐binding protein previously implicated in pre‐mRNA processing^26^ and identified as a genetic risk factor for multiple organ failure in acute pancreatitis,^27^ but its function in cancer has remained unknown. Our findings indicate that ZNF106 plays a key role in promoting M2 macrophage polarization by binding to the 3′UTR of IL‐6 mRNA, enhancing its stability, and promoting IL‐6 secretion and exosomal loading. IL‐6, a classic pro‐inflammatory cytokine, has been reported to participate in malignant progression in various tumours.^28^ We further found that M2 macrophage‐derived IL‐6 upregulates METTL3 expression in NSCLC cells by activating the JAK2/STAT3 signalling pathway. A similar mechanism has been reported in hepatocellular carcinoma.^21^ Our study extends this finding by revealing a new layer of regulation in tumour‐macrophage crosstalk: macrophage‐derived exosomal IL‐6 acts on NSCLC cells to activate the JAK2/STAT3 pathway, leading to METTL3 upregulation and forming a positive feedback regulatory loop. In vivo, using an orthotopic xenograft model, we validated the role of this loop in NSCLC progression. Macrophages treated with miR‑194‑5p mimics or IL‑6 knockdown reduced tumour growth and metastasis, with lower levels of METTL3, p‑JAK2, p‑STAT3, and Ki67. This validates the in vitro mechanisms and points to this loop as a potential therapeutic target in NSCLC.

Our findings suggest several potential therapeutic nodes. First, pharmacological inhibition of METTL3 using small‐molecule inhibitors (STM2457) may disrupt the positive feedback loop. STM2457 has demonstrated anti‐leukaemic activity by reducing m^6^A levels on leukaemogenic mRNAs,^29^ and has shown efficacy in oral squamous cell carcinoma^30^ as well as non‐small cell lung cancer.^31^ Second, exosomal delivery of miR‐194‐5p mimics can be developed to suppress ZNF106‐mediated M2 macrophage polarization. As natural miRNA carriers, exosomes—particularly engineered ones loaded with miRNA mimics—have shown clear anti‑tumour effects in preclinical models.^32^, ^33^ Furthermore, our study has confirmed that miR‐194‐5p is involved in macrophage polarization and immune regulation in NSCLC, providing a rationale for the use of engineered exosomes to deliver miR‐194‐5p mimics for NSCLC treatment. Third, ZNF106, as an RNA‐binding protein, might be amenable to targeted degradation via PROTAC technology. PROTACs have been employed to degrade BET proteins in castration‑resistant prostate cancer,^34^ and recent RNA‐based PROTACs have successfully targeted the RNA‐binding protein FMRP^35^ as well as nucleolin and SOX2 using aptamer‐based platforms.^36^ Based on the known RNA‐binding motif of ZNF106, an oligonucleotide‐based PROTAC could be rationally designed. Moreover, combining METTL3 inhibition with exosomal miR‐194‐5p mimic delivery may synergistically disrupt the positive feedback loop and repolarize the tumour microenvironment, potentially achieving superior anti‐tumour efficacy compared with either strategy alone. Future studies are warranted to evaluate these strategies in preclinical models.

Several limitations should be acknowledged. The commercially acquired tissue array lacked long‐term follow‐up data and comprehensive clinicopathological parameters (TNM stage, lymph node status), which precluded survival analysis and robust stage‐based correlations. Thus, the translational conclusions regarding prognostic value remain preliminary. Future prospective cohorts with full clinical annotations are needed to confirm the clinical importance of the METTL3/miR‑194‑5p/ZNF106 axis.

## 5 CONCLUSIONS

This study reveals a negative feedback loop between NFIC and METTL3 in NSCLC. This loop regulates exosomal miR‐194‐5p through m^6^A modification, thereby inducing M2 macrophage polarization. The polarized M2 macrophages further upregulate METTL3 expression in NSCLC cells via the ZNF106/IL‐6/JAK2/STAT3 axis, establishing a positive feedback circuit that promotes tumour progression. These results advance our understanding of immune microenvironment remodelling and lay a foundation for targeting this circuit therapeutically.

## AUTHOR CONTRIBUTIONS

Kesong Shi, Shu Fang, Mingyue Hao and Han Meng conceived and designed the study. Kesong Shi, Shu Fang, Mingyue Hao and Han Meng performed all the experiments. Kesong Shi, Shu Fang, Mingyue Hao, Han Meng, Yuhang Jiang, Qiwen Li, Jiao Liang, Xiaolu He, Yi Hu, Linling Zhou, Qianrun Wang, Qiyuan Zhuo and Ji Wu analysed the data. Kesong Shi, Shu Fang, Mingyue Hao and Han Meng wrote the paper. Linling Zhou, Qianrun Wang, Qiyuan Zhuo and Ji Wu edited and revised the paper. All authors read and approved the final manuscript.

## CONFLICT OF INTEREST STATEMENT

The authors declare no conflicts of interest.

## ETHICS STATEMENT

The study was conducted in accordance with the Declaration of Helsinki. For the human participant study, ethical approval was obtained from the Ethics Committee of Shanghai Outdo Biotech Company (Approval No. YB M‐05‐02), and written informed consent was obtained from all participants. All animal procedures were performed in strict accordance with the National Institutes of Health Guide for the Care and Use of Laboratory Animals and were approved by the Animal Ethics Committee of Hubei University of Medicine (Approval No. 03125110R).

## Supporting information



Supporting Information

Supporting Information

Supporting Information

Supporting Information

Supporting Information

Supporting Information

Supporting Information

Supporting Information

## Data Availability

The data supporting the findings of this study are available from the corresponding author upon reasonable request.
