# Supplementary figures and images for: Tumour‐macrophage crosstalk initiated by NFIC/METTL3 negative feedback loop via exosomal miR‐194‐5p promotes NSCLC progression
Source: Clin Transl Med. 2026 Jun 30;16(7):e70728. doi: 10.1002/ctm2.70728 (PMC13319397; doi:10.1002/ctm2.70728)

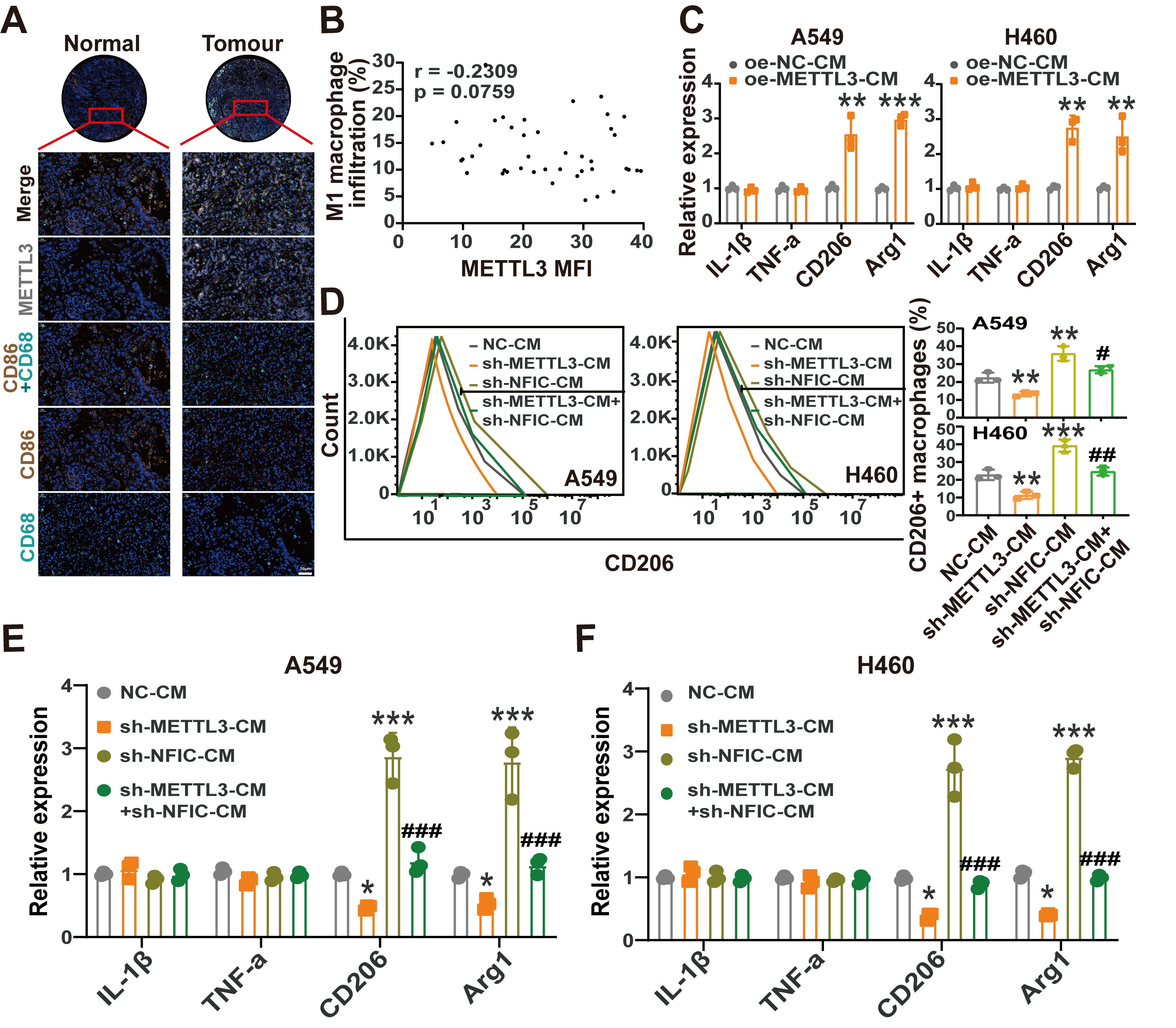

Supplement: Supplementary file 1 — Supporting Information [file CTM2-16-e70728-s002.tif]

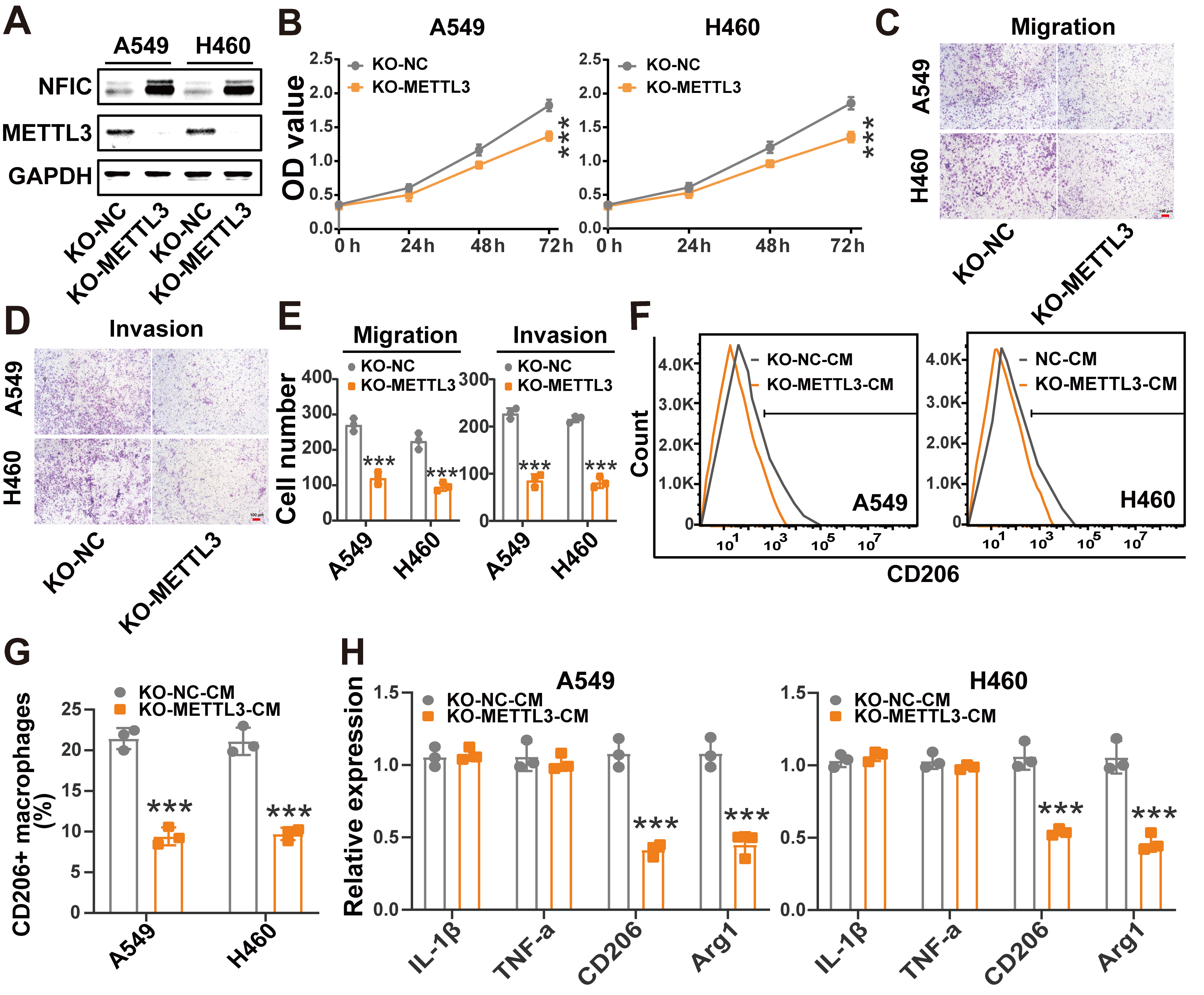

Supplement: Supplementary file 2 — Supporting Information [file CTM2-16-e70728-s008.tif]

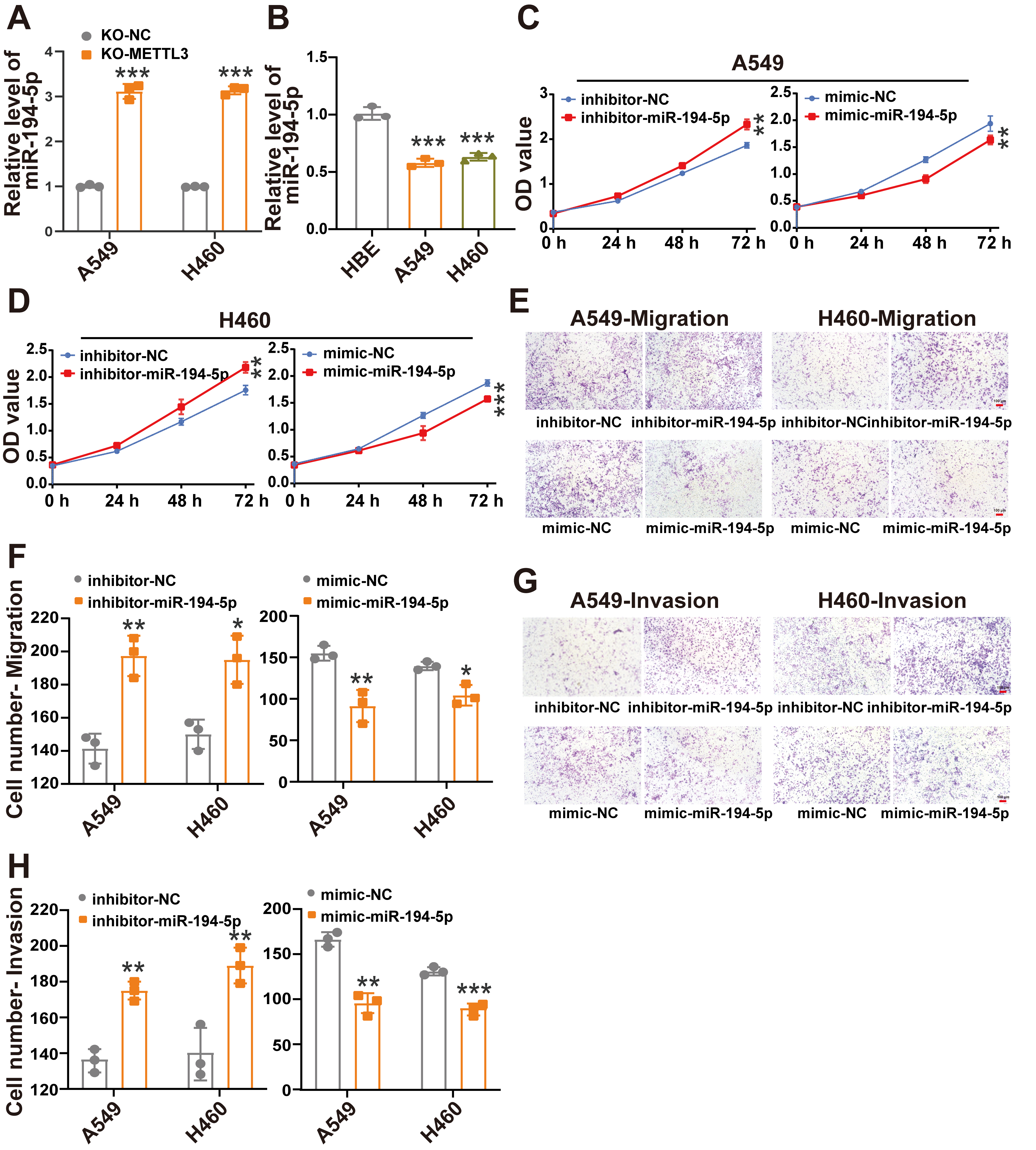

Supplement: Supplementary file 3 — Supporting Information [file CTM2-16-e70728-s004.tif]

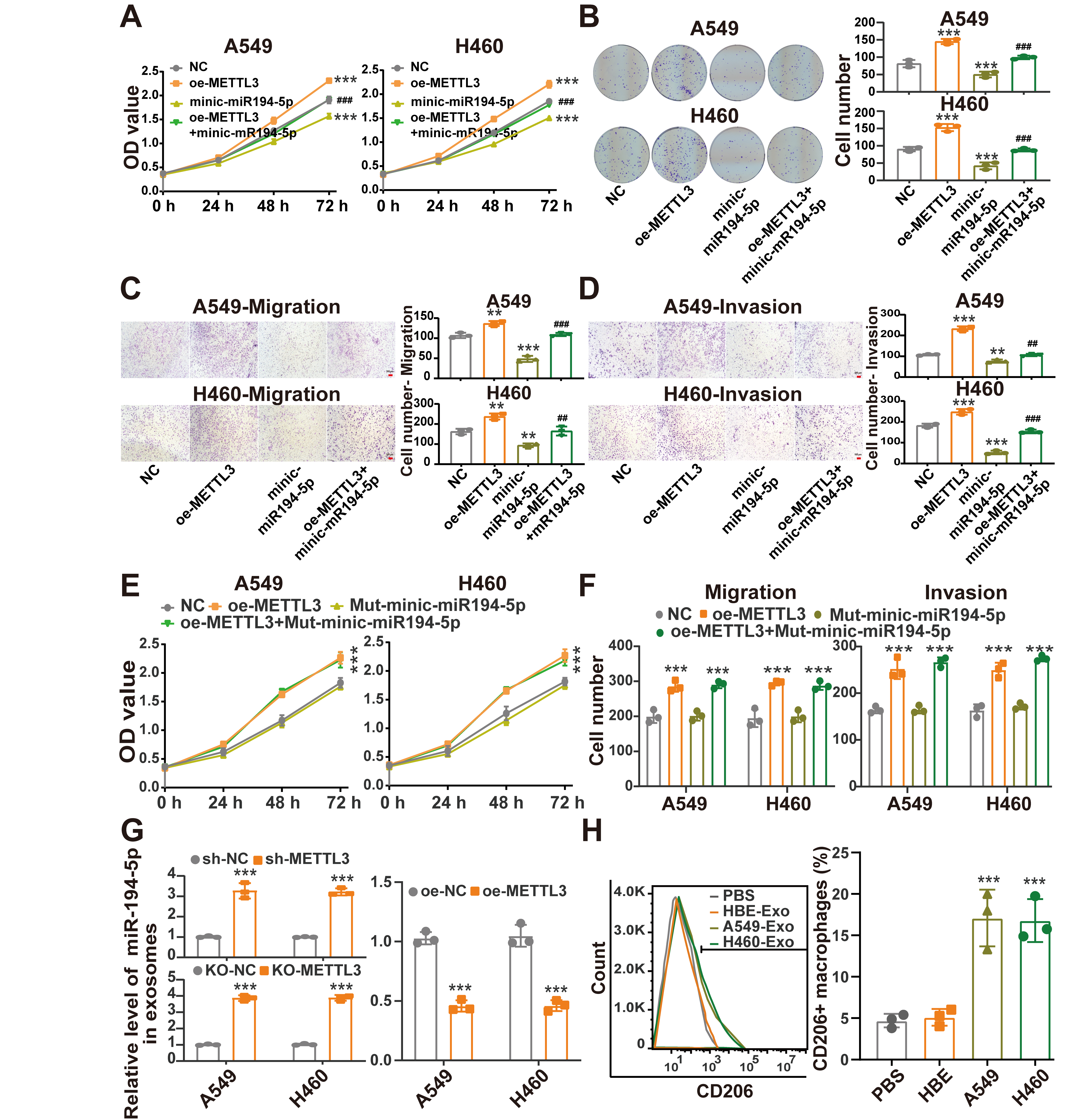

Supplement: Supplementary file 4 — Supporting Information [file CTM2-16-e70728-s005.tif]

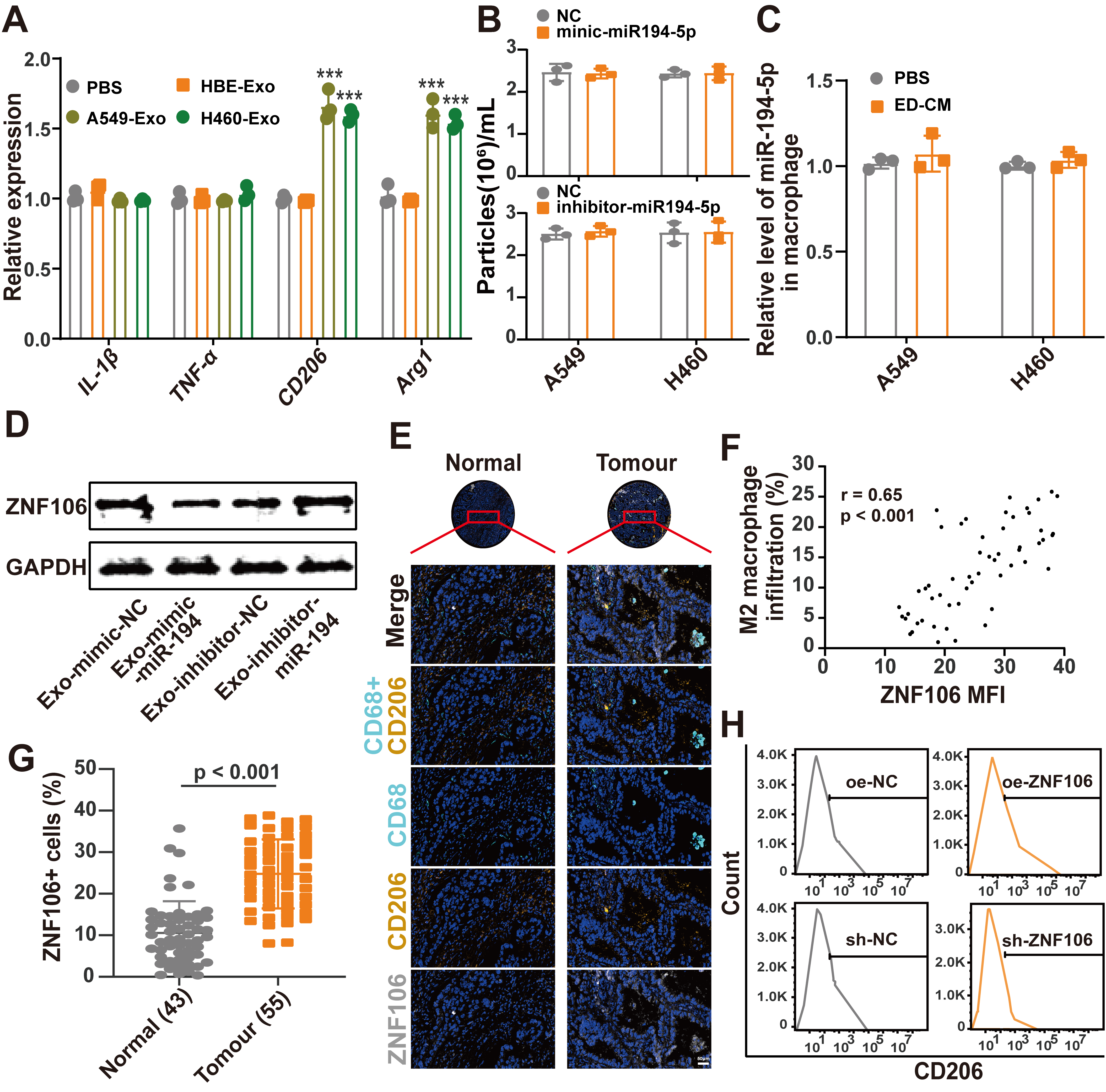

Supplement: Supplementary file 5 — Supporting Information [file CTM2-16-e70728-s001.tif]

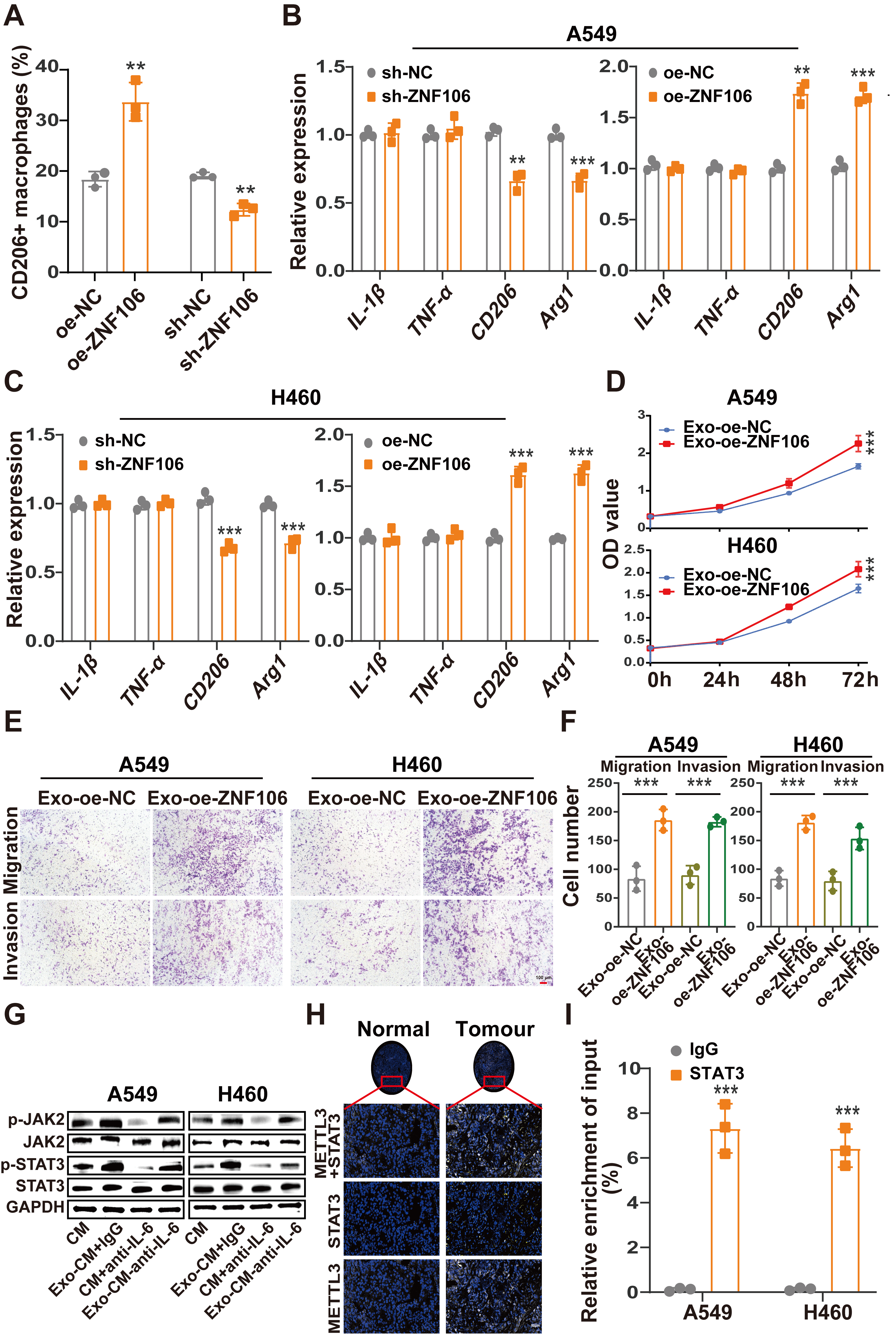

Supplement: Supplementary file 6 — Supporting Information [file CTM2-16-e70728-s003.tif]

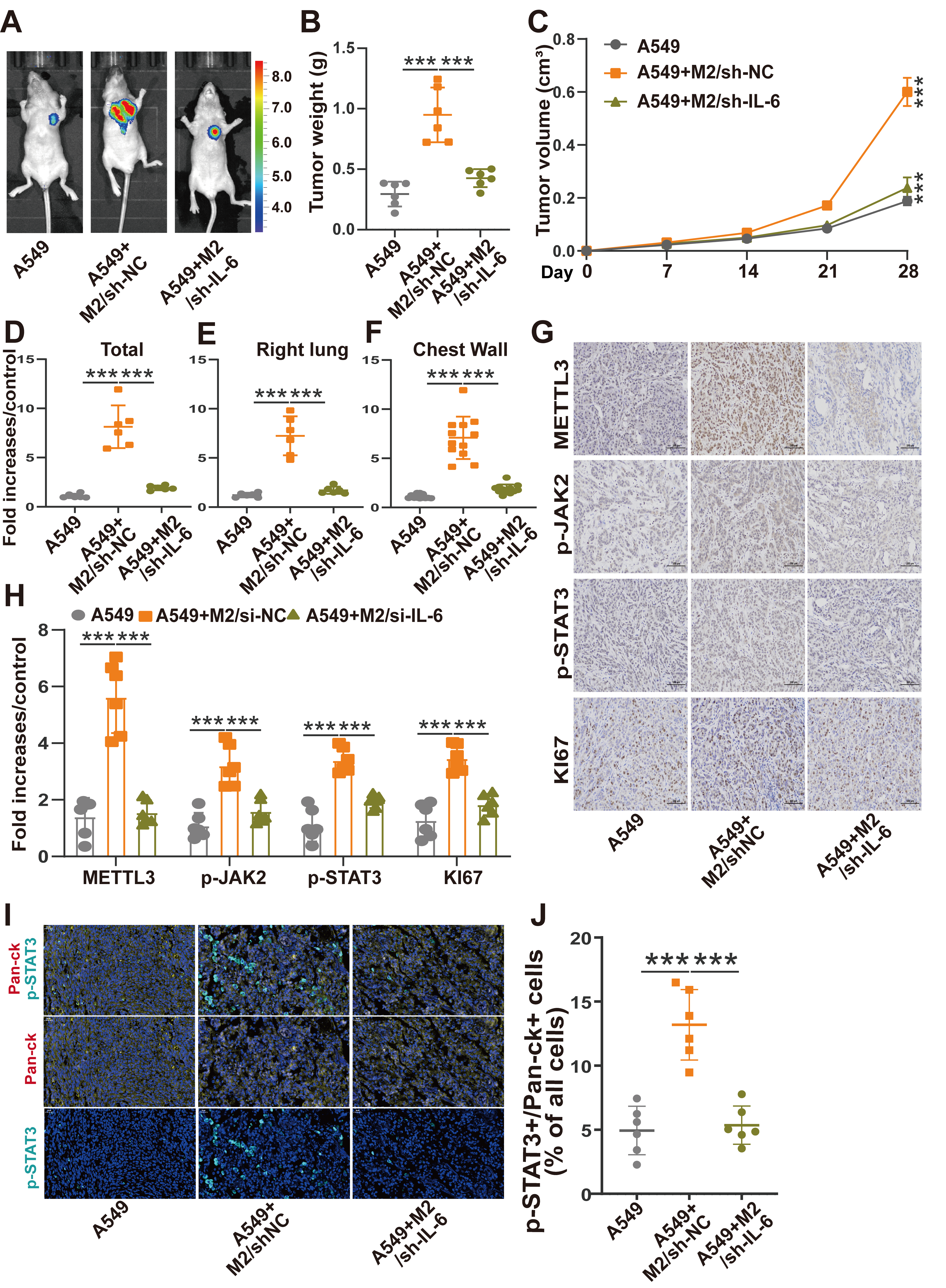

Supplement: Supplementary file 7 — Supporting Information [file CTM2-16-e70728-s006.tif]
