# Supplementary material for: Tumour‐macrophage crosstalk initiated by NFIC/METTL3 negative feedback loop via exosomal miR‐194‐5p promotes NSCLC progression
Source: Clin Transl Med. 2026 Jun 30;16(7):e70728. doi: 10.1002/ctm2.70728 (PMC13319397; doi:10.1002/ctm2.70728)
Supplement: Supplementary file 8 — Supporting Information [file CTM2-16-e70728-s007.docx]

Supporting Information

**Tumor-Macrophage Crosstalk Initiated by NFIC/METTL3 Negative Feedback Loop via Exosomal miR-194-5p Promotes NSCLC Progression**

**Short running title:** Tumor-Macrophage Crosstalk

Shu Fang^1,2,3†^, Mingyue Hao^1,2†^, Han Meng^1†^, Yuhang Jiang^1^, Qiwen Li^1^, Jiao Liang^1^, Xiaolu He^1^, Yi Hu^1^, Linling Zhou^1^, Qianrun Wang^1^, Qiyuan Zhuo^1^, Ji Wu^1^, Kesong Shi^1,2^*

^1^ Biomedical Research Institute, Hubei Key Laboratory of Wudang Local Chinese Medicine Research, Hubei University of Medicine, Shiyan 442000 China

^2^ Hubei Key Laboratory of Embryonic Stem Cell Research, Hubei University of Medicine, Shiyan 442000 China

^3^ School of Basic Medical Sciences, Hubei University of Medicine, Shiyan 442000 China

^†^ Shu Fang, Mingyue Hao, and Han Meng contributed equally to the work

* Correspondence: Kesong Shi

Biomedical Research Institute, Hubei Key Laboratory of Wudang Local Chinese Medicine Research, Hubei University of Medicine, Shiyan 442000 China; Hubei Key Laboratory of Embryonic Stem Cell Research, Hubei University of Medicine, Shiyan 442000 China.

Email address: sks.number1@hbmu.edu.cn

**Table S1. Information of NSCLC patients and tissue samples.**

| **Tissue Type** | **Tissue Code** | **Tumor/Surgery Organ** | **Sex** | **Smoking History** | **Pathological Type** | **Tumor Location** | **Tissue Code** |
| --- | --- | --- | --- | --- | --- | --- | --- |
| Cancer/Adjacent | E05A0835 | Lung | Male | Yes | Lung Adenocarcinoma | Lung | E05A0835 |
| Cancer/Adjacent | E05A0836 | Lung | Male | Yes | Lung Adenocarcinoma | Lung | E05A0836 |
| Cancer/Adjacent | E05A0837 | Lung | Male | Yes | Lung Adenocarcinoma | Lung | E05A0837 |
| Cancer/Adjacent | E05A0838 | Lung | Male | Yes | Lung Adenocarcinoma | Lung | E05A0838 |
| Cancer/Adjacent | E05A0840 | Lung | Male | Yes | Lung Adenocarcinoma | Lung | E05A0840 |
| Cancer/Adjacent | E05A0841 | Lung | Male | Yes | Lung Adenocarcinoma | Lung | E05A0841 |
| Cancer/Adjacent | E05A0843 | Lung | Male | Yes | Lung Adenocarcinoma | Lung | E05A0843 |
| Cancer/Adjacent | E05A0844 | Lung | Male | Yes | Lung Adenocarcinoma | Lung | E05A0844 |
| Cancer/Adjacent | E05A0845 | Lung | Male | Yes | Lung Adenocarcinoma | Lung | E05A0845 |
| Cancer/Adjacent | E05A0847 | Lung | Male | Yes | Lung Adenocarcinoma | Lung | E05A0847 |
| Cancer/Adjacent | E05A0848 | Lung | Male | Yes | Lung Adenocarcinoma | Lung | E05A0848 |
| Cancer/Adjacent | E05A0849 | Lung | Male | Yes | Lung Adenocarcinoma | Lung | E05A0849 |
| Cancer | E05A0839 | Lung | Male | Yes | Lung Adenocarcinoma | Lung | E05A0839 |
| Cancer | E05A0842 | Lung | Male | Yes | Lung Adenocarcinoma | Lung | E05A0842 |
| Cancer | E05A0846 | Lung | Male | Yes | Lung Adenocarcinoma | Lung | E05A0846 |
| Cancer/Adjacent | E05A0865 | Lung | Male | No | Lung Adenocarcinoma | Lung | E05A0865 |
| Cancer/Adjacent | E05A0866 | Lung | Male | No | Lung Adenocarcinoma | Lung | E05A0866 |
| Cancer/Adjacent | E05A0867 | Lung | Male | No | Lung Adenocarcinoma | Lung | E05A0867 |
| Cancer/Adjacent | E05A0868 | Lung | Male | No | Lung Adenocarcinoma | Lung | E05A0868 |
| Cancer/Adjacent | E05A0869 | Lung | Male | No | Lung Adenocarcinoma | Lung | E05A0869 |
| Cancer/Adjacent | E05A0871 | Lung | Male | No | Lung Adenocarcinoma | Lung | E05A0871 |
| Cancer/Adjacent | E05A0872 | Lung | Male | No | Lung Adenocarcinoma | Lung | E05A0872 |
| Cancer/Adjacent | E05A0873 | Lung | Male | No | Lung Adenocarcinoma | Lung | E05A0873 |
| Cancer/Adjacent | E05A0874 | Lung | Male | No | Lung Adenocarcinoma | Lung | E05A0874 |
| Cancer/Adjacent | E05A0875 | Lung | Male | No | Lung Adenocarcinoma | Lung | E05A0875 |
| Cancer/Adjacent | E05A0876 | Lung | Male | No | Lung Adenocarcinoma | Lung | E05A0876 |
| Cancer/Adjacent | E05A0878 | Lung | Male | No | Lung Adenocarcinoma | Lung | E05A0878 |
| Cancer/Adjacent | E05A0879 | Lung | Male | No | Lung Adenocarcinoma | Lung | E05A0879 |
| Cancer | E05A0870 | Lung | Male | No | Lung Adenocarcinoma | Lung | E05A0870 |
| Cancer | E05A0877 | Lung | Male | No | Lung Adenocarcinoma | Lung | E05A0877 |
| Cancer/Adjacent | E05A0896 | Lung | Female | Yes | Lung Adenocarcinoma | Lung | E05A0896 |
| Cancer/Adjacent | E05A0897 | Lung | Female | Yes | Lung Adenocarcinoma | Lung | E05A0897 |
| Cancer/Adjacent | E05A0900 | Lung | Female | Yes | Lung Adenocarcinoma | Lung | E05A0900 |
| Cancer/Adjacent | E05A0901 | Lung | Female | Yes | Lung Adenocarcinoma | Lung | E05A0901 |
| Cancer/Adjacent | E05A0902 | Lung | Female | Yes | Lung Adenocarcinoma | Lung | E05A0902 |
| Cancer/Adjacent | E05A0903 | Lung | Female | Yes | Lung Adenocarcinoma | Lung | E05A0903 |
| Cancer | E05A0895 | Lung | Female | Yes | Lung Adenocarcinoma | Lung | E05A0895 |
| Cancer | E05A0898 | Lung | Female | Yes | Lung Adenocarcinoma | Lung | E05A0898 |
| Cancer | E05A0899 | Lung | Female | Yes | Lung Adenocarcinoma | Lung | E05A0899 |
| Cancer | E05A0904 | Lung | Female | Yes | Lung Adenocarcinoma | Lung | E05A0904 |
| Cancer/Adjacent | E05A0914 | Lung | Female | No | Lung Adenocarcinoma | Lung | E05A0914 |
| Cancer/Adjacent | E05A0915 | Lung | Female | No | Lung Adenocarcinoma | Lung | E05A0915 |
| Cancer/Adjacent | E05A0916 | Lung | Female | No | Lung Adenocarcinoma | Lung | E05A0916 |
| Cancer/Adjacent | E05A0917 | Lung | Female | No | Lung Adenocarcinoma | Lung | E05A0917 |
| Cancer/Adjacent | E05A0918 | Lung | Female | No | Lung Adenocarcinoma | Lung | E05A0918 |
| Cancer/Adjacent | E05A0919 | Lung | Female | No | Lung Adenocarcinoma | Lung | E05A0919 |
| Cancer/Adjacent | E05A0920 | Lung | Female | No | Lung Adenocarcinoma | Lung | E05A0920 |
| Cancer/Adjacent | E05A0922 | Lung | Female | No | Lung Adenocarcinoma | Lung | E05A0922 |
| Cancer/Adjacent | E05A0923 | Lung | Female | No | Lung Adenocarcinoma | Lung | E05A0923 |
| Cancer/Adjacent | E05A0925 | Lung | Female | No | Lung Adenocarcinoma | Lung | E05A0925 |
| Cancer/Adjacent | E05A0926 | Lung | Female | No | Lung Adenocarcinoma | Lung | E05A0926 |
| Cancer/Adjacent | E05A0927 | Lung | Female | No | Lung Adenocarcinoma | Lung | E05A0927 |
| Cancer | E05A0913 | Lung | Female | No | Lung Adenocarcinoma | Lung | E05A0913 |
| Cancer | E05A0921 | Lung | Female | No | Lung Adenocarcinoma | Lung | E05A0921 |
| Cancer | E05A0924 | Lung | Female | No | Lung Adenocarcinoma | Lung | E05A0924 |

**Table S2. gRNA sequences for CRISPR/Cas9-mediated METTL3 knockout**

| **sgRNA** | **Sequence** |
| --- | --- |
| sgMETTL3-F | CACCGGGGCTGTCACTACGGAAGGT |
| sgMETTL3-R | AAACACCTTCCGTAGTGACAGCCCC |

**Table S3. The sequences of shRNA**

| **Target** | **Sequence (5’-3’)** |
| --- | --- |
| shRNA - control | TTCTCCGAACGTGTCACGT |
| shRNA - METTL3 | GCTGCACTTCAGACGAATTAT |
| shRNA – ZNF106 | TTCAGAGGCTACTTATGCTC |

**Table S4. Primer sequences of target genes**

| **Target** | **Sequence (5’-3’)** |
| --- | --- |
| *GAPDH* | F:5’-GCACCGTCAAGGCTGAGAAC-3’ |
|  | R:5’-TGGTGAAGACGCCAGTGGA-3’ |
| *NFIC* | F:5'-TGGCGGCGATTACTACACTTCG-3' |
|  | R:5’-GGCTGTTGAATGGTGACTTGTCC-3’ |
| *METTL3* | F:5’-CAAGCTGCACTTCAGACGAA-3’ |
|  | R:5’-GCTTGGCGTGTGGTCTTT-3’ |
| *IL-1β* | F:5’- CAGAAGTACCTGAGCTCGCC -3’ |
|  | R:5’- AGATTCGTAGCTGGATGCCG -3’ |
| *TNF-a* | F: 5’- TCTTCTCGAACCCCGAGTGA -3’ |
|  | R: 5’- ATGAGGTACAGGCCCTCTGA -3’ |
| *CD206* | F: 5’- CGTTCCTTTGGACGGATGGA -3’ |
|  | R: 5’- CCTCGTTTACTGTCGCAGGT -3’ |
| *Arg-1* | F:5’- ACTTAAAGAACAAGAGTGTGATGTG -3’ |
|  | R:5’- CATGGCCAGAGATGCTTCCA -3’ |
| *ZNF106* | *F:5’- ACTGTCTGTCATCAAGCGCA -3’* |
|  | *R:5’- AGCTGGTCAACCTGAGAACG -3’* |
| *VPS13B* | F:5’-GCAGGACTGACGTCTTTGGA -3’ |
|  | R:5’-TCCAGCATGTGGGGAGTTTC-3’ |
| *U6* | F:5’- CTCGCTTCGGCAGCACA -3’ |
|  | R:5’- AACGCTTCACGAATTTGCGT -3’ |

**Table S5. Antibody Information**

| **Target Protein** | **Predicted Dilution** | **Recommended Supplier** | **Catalog numbers** |
| --- | --- | --- | --- |
| NFIC | WB: 1:1000 | Proteintech, China | 68318-1-Ig |
| METTL3 | WB: 1:1000  IHC: 1:500 | Abcam, Cambridge, UK | ab195352 |
| Arginase-1 | WB: 1:5000 | Proteintech, China | 66129-1-Ig |
| CD68 | IHC: 1:1000  FC: 1:100 | Proteintech, China | 66231-2-Ig |
| CD68 | FC: 5 ul per 10^6 cells in 100 μl suspension | Proteintech, China | PE-FcA65593 |
| CD86 | IHC: 1:1000  FC: 1:100  IF/ICC: 1:200 | Proteintech, China | 31449-1-AP |
| CD206 | WB: 1:5000  FC: 1:100  IHC: 1:5000 | Proteintech, China | 60143-1-Ig |
| ZNF106/ ZFP106 | WB: 1:1000; IF: 1:200 | Abmart, China | PJP09673 |
| IL-6 | WB: 1:1000; ELISA: 1:400; | Proteintech, China | 66146-1-Ig |
| IL-10 | WB: 1:1000; ELISA: 1:400; | Proteintech, China | 60269-1-Ig |
| TGF-β | WB: 1:1000; ELISA: 1:400; | Proteintech, China | 81746-2-RR |
| CCL1 | WB: 1:1000; ELISA: 1:400; | Abmart, China | PK13291 |
| VEGF | WB: 1:1000; ELISA: 1:400; | Abmart, China | M023285 |
| STAT3 | WB: 1:1000; IF: 1:200 | Abmart, China | T55292 |
| p-STAT3 | WB: 1:1000; IF: 1:200 | Abmart, China | TC52210 |
| JAK2 | WB: 1:1000; IF: 1:200 | Abmart, China | TA6022 |
| p-JAK2 | WB: 1:1000; IF: 1:200 | Abmart, China | T56570 |
| Pan- Cytokeratin | WB: 1:1000  IHC: 1:2000  IF: 1:2000 | Servicebio, China | GB152053 |
| Anti-Mouse | 1:5000 | Proteintech, China | SA00001-1 |
| Anti-Rabbit | 1:5000 | Proteintech, China | SA00001-2 |

**MATERIALS AND METHODS**

**Cell Culture**

The A549 and H460 NSCLC cell lines, as well as the HBE normal human lung epithelial cell line, were obtained from the American Type Culture Collection (ATCC) in Manassas, USA. The THP-1 cell line was also obtained from ATCC. A549 cells were maintained in DMEM with 10% fetal bovine serum (Gibco, USA). H460, HBE, and THP-1 cells were cultured in RPMI 1640 medium (VivaCell, Shanghai, China). Cells were incubated at 37°C with 5% CO₂ in a humidified Thermo Fisher Scientific incubator (USA).

**RNA Immunoprecipitation (RIP)**

RIP is a technique used to analyze RNA-protein interactions. This method involves the immunoprecipitation of RNA-binding proteins along with their associated RNA molecules, allowing for the identification and characterization of RNA sequences that interact with specific proteins. RNA-protein binding complexes were detected using RIP assays with the Magna RIP™ RNA-Binding Protein Immunoprecipitation Kit (Millipore, USA). Cells were lysed in lysis buffer containing protease inhibitors and RNase inhibitors. Magnetic beads were pre-incubated with the target primary antibody or negative control IgG for 45 min. The cell lysate was incubated with antibody-conjugated beads overnight at 4°C. After digestion of the RNA–protein complexes with proteinase K, the immunoprecipitated RNA was purified and subjected to qRT‑PCR.

**Dual-Luciferase Reporter Assay**

Beyotime (Shanghai, China) synthesized wild‑type and mutant fragments of the NFIC 3′UTR (containing the predicted m⁶A motif) as well as wild‑type and mutant sequences of miR‑194‑5p and the ZNF106 3′UTR (with predicted binding sites). These were cloned into the pmirGLO luciferase reporter vector (Promega, WI, USA). For reporter assays, cells plated in 6‑well plates were cultured overnight and then co‑transfected with the constructs. Luciferase activity was measured at 48 h post‑transfection using the Dual‑Luciferase Reporter Assay System (Promega).

**Cell Proliferation Assays**

Cell proliferation was assessed using the CCK-8 assay (G4103, Servicebio, Wuhan, China). A549 and H460 cells were plated into 96‑well plates at 5 × 10^3^ cells per well. After the indicated treatments, cell viability was measured every 24 h over 3 days using CCK-8 reagent (2 h incubation) and reading absorbance at 450 nm on a microplate reader. For colony formation assays, cells (800/well) were plated in 6‑well plates and cultured for ~12 days (medium changed every 2 d). Colonies were then fixed (4% paraformaldehyde, 10 min), stained (0.1% crystal violet, 10 min), and manually counted. For EdU assays, cells (2 × 10⁵/well) seeded in 6‑well plates received EdU reagent (C0075s, Beyotime) for 2 h after treatment, followed by fixation with 4% paraformaldehyde and fluorescent staining per the manufacturer’s protocol. ImageJ was used to quantify EdU‑positive cells.

**Cell Migration and Invasion Assays**

A549 and H460 cells were suspended in serum-free medium and seeded into the upper chambers at a density of 5 × 10^4^ cells per well. The lower chambers were filled with medium containing 10% fetal bovine serum. Following 24 h at 37 °C, migrated cells on the lower membrane surface were fixed (4% paraformaldehyde), stained (0.1% crystal violet), and manually enumerated under a microscope. For invasion assays, the upper chambers were pre-coated with Matrigel (356234, Corning, Acton, USA), and remaining procedures were performed as described for the migration assay.
